# Quality by Design-Guided Development of Hydrogel-Forming Microneedles for Transdermal Delivery of Enfuvirtide

**DOI:** 10.1021/acsami.5c00499

**Published:** 2025-03-10

**Authors:** Huanhuan Li, Lalitkumar K. Vora, Qonita Anjani, Abraham M. Abraham, Yilin Cong, Natalia Moreno-Castellanos, Ester Ballana, Eva Riveira Muñoz, Maria Nevot, Ryan F. Donnelly

**Affiliations:** † School of Pharmacy, Queen’s University Belfast, BelfastBT9 7BL, U.K.; ‡ Basic Science Department, Faculty of Health, Universidad Industrial de Santander, Bucaramanga 680001, Colombia; § AIDS Research Institute—IrsiCaixa, 08916 Badalona, Spain

**Keywords:** enfuvirtide, hydrogel-forming microneedles, quality by design, design of experiment, critical material attributes, critical process parameters, critical quality attributes, transdermal drug delivery

## Abstract

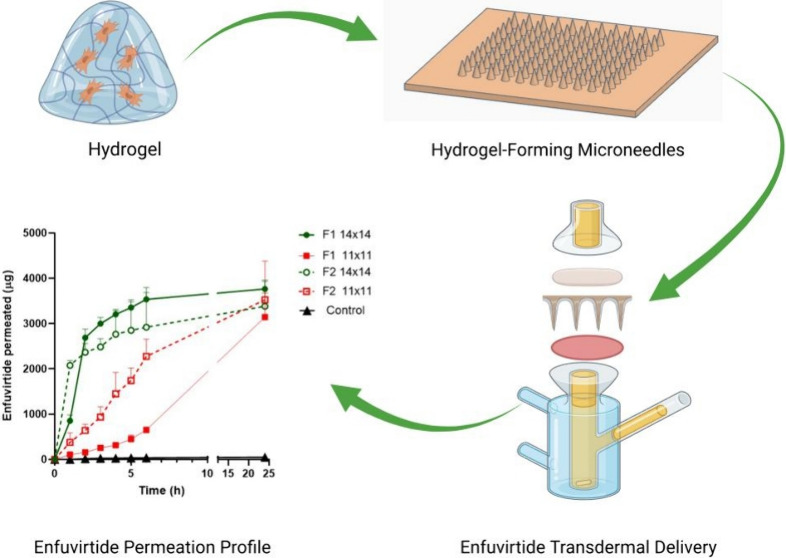

Enfuvirtide, the inaugural biomimetic fusion inhibitor of HIV-1, has exhibited remarkable antiviral efficacy when administered in conjunction with an optimized antiretroviral regimen. Nonetheless, the high incidence (98%) of injection site reactions associated with twice-daily subcutaneous administration severely compromises patient adherence and long-term therapeutic outcomes. This study proposes hydrogel-forming microneedles (MNs) as a minimally invasive and painless modality for the transdermal delivery of this therapeutic peptide. Leveraging a rigorous Quality by Design (QbD) framework, this investigation systematically delineated the critical material attributes (CMAs) and critical process parameters (CPPs) of the hydrogel formulation, mapping their influence on the critical quality attributes (CQAs) of MNs to achieve a meticulously defined quality-target product profile (QTPP). The optimized MN formulation, achieving a desirability index of 0.871, was validated through comprehensive design space and feasibility analyses, demonstrating superior predictive accuracy and mechanical integrity. Ex vivo permeation studies elucidated the sustained and controlled release kinetics of enfuvirtide via MNs fabricated from the optimized formulation, attaining a maximum permeation of 36.26% using 11 × 11 molds, compared to 28.45% permeation observed with the control system over 24 h. Furthermore, the system’s favorable swelling kinetics and enhanced viscoelastic properties significantly augmented its delivery performance relative to conventional approaches. This study not only establishes hydrogel-forming MNs as an innovative and efficacious delivery platform for enfuvirtide but also presents a robust, systematic methodology for MN development, offering transformative potential for broader pharmaceutical applications and therapeutic paradigms.

## 1 Introduction

As the first biomimetic fusion inhibitor of human immunodeficiency virus-1 (HIV-1), enfuvirtide has demonstrated remarkable antiviral activity and a favorable safety profile when used in combination with an optimized background antiretroviral regimen.^1−4^ However, its clinical adoption is significantly hindered by the requirement for twice-daily subcutaneous injections (90 mg), which lead to injection site reactions (ISRs) in approximately 98% of patients.^5−8^ These ISRs often result in inflammation, tissue damage, and subsequent treatment discontinuation.^9,10^ To mitigate these issues, alternative delivery strategies have been explored. The Biojector, a needle-free jet injector, was developed to administer enfuvirtide intradermally using high-pressure liquid propulsion.^11^ However, its clinical utility is limited by drug wastage, variable bioavailability, and transient pain when injections occur near nerves.^12,13^ Similarly, long-acting formulations, including poly(lactic-*co*-glycolic) acid (PLGA) microparticles,^14^ polymer–lipid hybrid nanoparticles,^15^ poly(ethylene glycol)-covalent (PEGylated) enfuvirtide^16^ and in situ self-gelling systems,^17^ have been investigated. While promising, these approaches remain in early development and have not yet overcome key challenges, such as precise dosing control and patient adherence.

Microneedles (MNs) represent a minimally invasive alternative to traditional injections with potential advantages over other needle-free technologies. Unlike jet injectors, which require high pressure and may cause discomfort, MNs painlessly penetrate the *stratum corneum*, forming microchannels that facilitate controlled drug diffusion (Figure S1). Hydrogel-forming MNs’ unique ability to swell upon exposure to interstitial fluid enables sustained drug release, while their biocompatible nature minimizes residual waste.^18−20^ Additionally, MNs bypass first-pass metabolism and can improve patient compliance by enabling self-administration, making them particularly valuable for chronic therapies like enfuvirtide.^21^ Studies have shown that MNs are capable of delivering peptides with a range of molecular weights (Table S1). Despite their promise, MN technology still requires refinement, particularly in terms of optimizing formulation parameters to ensure consistent drug delivery and manufacturability.

Quality by Design (QbD), first introduced by Joseph M. Juran and later incorporated into regulatory guidelines such as the International Council on Harmonisation of Technical Requirements for Registration of Pharmaceuticals for Human Use (ICH) Q8 (R2), ICH Q9 and ICH Q10, offers a systematic approach to pharmaceutical development by emphasizing statistical principles, risk management, and process control.^22−26^ The Design of Experiments (DoE) methodology has been widely applied in pharmaceutical research to identify critical parameters influencing drug formulation and manufacturing.^27^ A search of Scopus using the keywords “design of experiments” and “pharmaceutical” retrieved over 1400 studies, underscoring its broad application in drug development (Table S2). However, its implementation in optimizing hydrogel-forming MNs remains limited.

**Figure 1:**
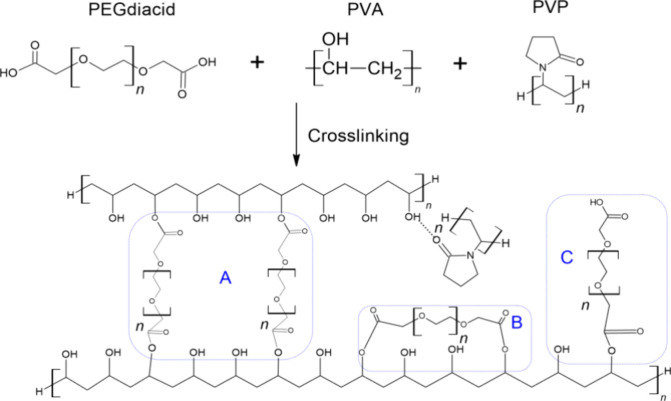
Proposed cross-linking reactions in PVA/PVP/PEGdiacid hydrogels. (A) Both carboxyl groups of two PEGdiacid molecules react with hydroxyl groups from different PVA chains, forming interchain cross-links. (B) Both carboxyl groups of a single PEGdiacid molecule react with hydroxyl groups from the same PVA chain, creating intrachain cross-links. (C) A single carboxyl group of PEGdiacid reacts with a hydroxyl group from PVA, resulting in partial cross-linking.

**Figure 2:**
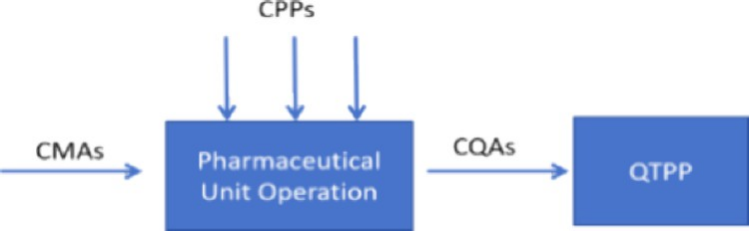
Illustrative correlation of the inputs and outputs within the pharmaceutical product development process for MNs, modified from ref (22).

In response to the growing need for regulatory clarity and standardization, the Microneedle Array Patch (MAP) Regulatory Working Group (RWG) released a White Paper in 2024 outlining 20 critical quality attributes (CQAs) essential for guiding innovation in MN technology.^28^ This framework provides a structured basis for improving formulation design, manufacturing consistency, and clinical translation. In this study, hydrogel-forming MNs were fabricated using poly(vinyl alcohol) (PVA) as the primary polymer,^29^ cross-linked with poly(ethylene glycol) bis(carboxymethyl) ether (PEGdiacid) to enhance flexibility,^30^ and reinforced with poly(vinylpyrrolidone) (PVP) for improved mechanical strength.^31^ The proposed cross-linking reactions are shown in Figure 1. By systematically applying DoE principles, we identified the critical material attributes (CMAs) and critical process parameters (CPPs) essential for optimizing MN formulation. The CMAs and CPPs were subsequentially associated with the CQAs of MNs for the assurance of the quality target product profile (QTPP) by establishing mathematical relationships between the inputs and outputs of the process systematically (Figure 2). The physicochemical properties, biocompatibility, and transdermal permeation efficacy of these MNs were subsequently evaluated alongside the antiviral activity of enfuvirtide-loaded drug reservoirs against MT4 cell lines.

This work aims to advance the clinical feasibility of hydrogel-forming MNs as a pain-free, patient-friendly alternative for enfuvirtide delivery, addressing key limitations associated with conventional injections and other noninvasive delivery strategies. By integrating QbD principles into MN design, we seek to establish a robust framework for ensuring the reliability, reproducibility, and regulatory compliance of this innovative drug delivery platform.

## 2 Experimental Study

### 2.1 Materials

Enfuvirtide was obtained from Roche (Basel, Switzerland). Plasdone/poly(vinylpyrrolidone) (PVP K-29/32, *M*_W_ 10,000) was gifted by Ashland (Kidderminster, UK). Poly(vinyl alcohol) (PVA 87–89% hydrolyzed *M*_W_ 85,000–124,000), citric acid monohydrate, acetonitrile for high-performance liquid chromatography (HPLC), poly(ethylene glycol) bis(carboxymethyl) ether (PEGdiacid, average *M*_n_ 250), phosphate buffered saline tablets (PBS), zidothymidine (AZT), and 3-(4,5-dimethylthiazol-2-yl)-2,5-diphenyltetrazolium bromide (MTT) were purchased from Sigma-Aldrich (Dorset, UK). Water for buffer and HPLC was obtained from ELGA Purelab Flex 2 (Vivendi Water System Ltd., Bucks, UK) ultrapure water purification system. Dermatome and full-thickness skin samples were excised from unborn piglets and stored at −20 °C before use. Parafilm M was supplied by Bemis Company Inc. (Soignies, Belgium). Mannitol was obtained from Roquette (Lestrem, France). Gelatin was purchased from Mr.P Ingredients (York, UK). MT4 cells (ECACC 08081402) were cultured in RPMI 1640 medium (Gibco, Waltham, MA, USA) supplemented with 10% fetal calf serum (FCS) (Gibco, Waltham, MA, USA) and 1% penicillin/streptomycin. Solution 13 (30 μg/mL acridine orange (AO) and 100 μg/mL 4,6-diamidino-2-phenylindole (DAPI) in water) was purchased from ChemoMetec (Allerod, Denmark). HIV-1 NL4–3 virus was obtained from the MRC Centre for AIDS Reagents (London, UK). The viral stock of the fully replicative NL4–3 clone was grown in lymphoid MT-4 cells.

### 2.2 Design of Experiments for Hydrogel-Forming MN Formulations

Preliminary studies indicated that the cross-linker PEGdiacid amount (% w/w), cross-linking time (min), and cross-linking temperature (°C) were the main factors of the manufacturing process that affect the CQAs of the hydrogel including equilibrium water content (EWC), gel fraction (GF) and drug diffusion percentage across the swollen hydrogel. Therefore, a 3-factor 3-level and 3-response central composite design-response surface methodology (CCD-RSM) was adopted in this case to systemically investigate the influence of the three critical formulation variables on the hydrogel. The experimental range of the factors was determined from exploratory test results and the feasibility of preparing the hydrogel at extreme values, where the compositions of PVA and PVP were fixed at 15 and 10% (w/w). Factors with levels and responses with goals are presented in Table 1. A total of 19 tests (Table S3) were performed according to the suggested results from Design Expert version 12.0.3 software (Stat-Ease Inc., Minneapolis, Minnesota, USA) to evaluate the effects of three independent variables (*X*1, ratio of PEGdiacid, *X*2, cross-linking temperature, and *X*3, cross-linking time) on the swelling property of the hydrogel, the gel fraction, and the enfuvirtide diffusion percentage. All of the formulations used in these experiments were prepared in triplicate.

**Table 1:** Levels of the Factors and the Goal of the Responses in the Central Composite Design

	level			response	goal
factors	–1	0	+1		
X1= cross-linker ratio (% w/w)	2.25	6.19	10	R1 = EWC (%)	in range
X2 = temperature (°C)	80	116.58	150	R2 = gel fraction (%)	in range
X3 = time (min)	20	48.95	80	R3 = diffusion (%)	maximize

### 2.3 Fabrication of Hydrogel-Forming Films

To facilitate the evaluation of hydrogels, square-shaped films with a dimension of 1 × 1 × 0.5 cm were produced. The hydrogel-forming films were made of aqueous blends of polymers and cross-linkers according to the composition listed in Table S3. Stock solutions of PVA (25% w/w) and PVP (40% w/w) were prepared by dissolving accurately weighed powder in deionized water and mixing until homogeneous. For hydrogel fabrication, the required masses of the ingredients were weighed, followed by the addition of deionized water to reach 100% weight. The blends were mixed until they were homogeneous. To remove air bubbles, the blend was centrifuged at 3500 rpm for 15 min using an Eppendorf 5804 R centrifuge (Eppendorf UK Ltd., Stevenage, UK). Subsequently, 0.45 mg of the blend was weighed into the mold with a size of 1 × 1 × 0.5 cm and then centrifuged at 3500 rpm for 15 min to form a layer of polymeric solution inside the mold, after which air bubbles were removed. The formulations were dried at ambient temperature for 48 h and followed by subjecting to cross-linking at the conditions listed in Table S3.

### 2.4 Swelling Studies of Hydrogel-Forming Films

The swelling studies were conducted by immersing the hydrogel-forming film obtained in 15 mL of PBS (pH 7.4) for 24 h at ambient temperature. Specifically, the hydrogel was weighed, and the mass was recorded as the initial weight (*m*_0_). The hydrogel was subsequently placed in a container with 15 mL of PBS. At a predetermined time point, the hydrogel was removed, gently dabbed with filter paper on the surface to remove excess buffer, weighed, and recorded as *m_t_*. The equilibrium water content (EWC) was calculated via eq 1. The gel fraction (GF) was obtained through the same process as the EWC, except the PBS was replaced with deionized water to exclude the weight from the salt. After 24 h, the swollen hydrogel was dried at 80 °C for 24 h and then weighed, and the weight was recorded as *m*_d_. GF was calculated via eq 2.
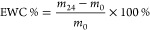
1
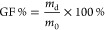
2

### 2.5 Enfuvirtide Diffusion Studies across Hydrogels

Horizontal diffusion cells (PermeGear Inc., New Jersey, USA) were employed for diffusion studies of enfuvirtide through pre-equilibrated hydrogels. The hydrogel film was swelled in PBS 24 h prior to the study to achieve equilibrium and was placed between the donor and the receptor compartments. The two compartments were secured with Parafilm to ensure that the hydrogel covered the holes of the compartments (Figure S2). Then, the apparatus was placed on a magnetic stirring station and warmed with a 37 °C circular water bath (Julabo UK Ltd., Stamford, UK). The donor chamber was filled with 3 mL of a 1 mg/mL enfuvirtide solution prepared in 50% ethanol (ethanol in deionized water, v/v). This solvent system was chosen to enhance the solubility of enfuvirtide and to ensure its stability during the experiment.^32^ The receptor chamber was filled with 3 mL of 50% ethanol, which was drained at the specified time interval and immediately replaced with the same volume of 50% ethanol. The drug concentrations in the samples were then analyzed via methods described in a previous publication^32^ and recorded as *C_t_* (μg/mL). The drug diffusion percentage was calculated via eq 3.
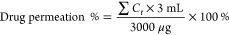
3

### 2.6 Rheological Measurements of Swollen Hydrogels

The viscoelastic properties of the swollen hydrogels were evaluated with a modular compact rheometer MCR 102e (Anton Paar, Graz, Austria) equipped with a parallel plate with a diameter of 25 mm (PP25/P2) and a 60 mm diameter-stainless steel inset (PP60/SS), as depicted in Figure S3. Amplitude sweeps and frequency sweeps were conducted for dynamic shear oscillation measurements of the storage modulus (*G*′), loss modulus (*G*″), and linear viscoelastic region (LVER) limits of the hydrogels. For the amplitude sweep mode, the angular frequency (ω) was maintained at 2.5 rad/s, and 51 data points were collected from the shear strain (γ) within 0.1–1000%. For frequency sweeps, 41 points of mechanical spectra were recorded in constant strain mode, with a low deformation of 0.5% maintained over the frequency range of 1–100 rad/s. The plate-to-plate distance was set according to the thickness of the hydrogel, and the temperature was set at 37 °C for both modes.

### 2.7 Optimization of MN Formulations

The results of the responses from the performed experiments were filled in the software according to the formulation. The relationships between response variables and formulation factors in all model formulations were assessed via Design-Expert software. Statistical analyses, including stepwise linear regression, residual analysis, and analysis of variance (ANOVA), were carried out. Only terms with significance levels (*p* < 0.05) were incorporated into the final equations. The models, featuring linear, quadratic, and special cubic components, were evaluated. The selection of the most suitable mathematical model was based on comparisons of statistical parameters such as the coefficient of variation (CV), the multiple correlation coefficient (*R*^2^), and the adjusted multiple correlation coefficient (adjusted *R*^2^), as determined by Design-Expert software. Differences in significance were examined via Student’s *t-*test and one-way ANOVA at a probability level of 0.05. Response surface plots were also generated by the software for graphically analyzing the effects of the independent variables on the response. The formulation with the highest desirability was selected as the optimal solution recommended by the software, and this was then used to fabricate hydrogels and MNs after model validation.

#### 2.7.1 Chemical and Thermal Analysis of the Hydrogels

Fourier-transform infrared spectroscopy (FTIR) analysis and thermal analysis were used to characterize the hydrogel before and after cross-linking. The absorbance of the hydrogel materials in the infrared region was measured via a PerkinElmer Spectrum Two FT-IR Spectrometer (PerkinElmer, Buckinghamshire, UK) coupled with a Pike MIRacle ATR attachment (Pike Technologies, Wisconsin, USA). Individual samples were mounted into the ATR diamond head and secured via the instrument’s screw. The absorbance at 750–4000 cm^–1^ was subsequently measured, with 32 scans of each sample. The acquired spectra were processed and analyzed using Spectro-IR software. The results (after background correction) were normalized and plotted as transmittance versus wavenumber via Origin9.

The thermal properties of the hydrogels and their compositions were investigated via differential scanning calorimetry (DSC) and thermogravimetric analysis (TGA) (TA Instruments, New Castle, USA). For DSC, 5–10 mg samples were weighed and placed in a sealed aluminum crucible (aluminum pan and lid). The sample was then placed on top of the heating table, side by side with the reference crucible. The DSC was run under nitrogen flow conditions from 25 to 400 °C with a heating rate of 10 °C/min. Heatmaps were created and analyzed using TA Instruments Universal Analysis 2000 software. For TGA, 5–10 mg of the sample was placed into an open aluminum crucible and placed on the autoloader of the TGA system. The sample was heated in ramp mode, and the weight change was recorded. Weight loss percentages were analyzed and plotted using the same software used for DSC.

### 2.8 Biocompatibility Assessment of the Hydrogels

The biocompatibility of hydrogels was assessed using HaCaT cells, a spontaneously immortalized human keratinocyte line, where cell viability was assessed using the MTT assay, live/dead staining, and a cell proliferation assay. HaCaT cells were seeded and incubated with hydrogel samples in complete Dulbecco’s modified Eagle’s medium (DMEM) for 72 h at 37 °C in a 5% CO_2_ atmosphere. The MTT assay was performed as previously described.^33^ Briefly, the culture medium was replaced with an MTT solution (0.5 mg/mL in DMEM), and after 5 h, the supernatant was removed. Dimethyl sulfoxide (DMSO, 200 μL) was then added, followed by gentle shaking for 15 min to dissolve the formazan crystals. Optical absorbance was measured at 570 nm via a Synergy H1 microplate reader (Agilent, Santa Clara, United States). Cells cultured without hydrogels but medium served as positive controls, while 1% Triton X-100 was used as a negative control. To further assess the cell viability, live/dead staining was conducted. After 72 h of incubation, the cells were incubated at room temperature for 5 min with 5 μg/mL calcein AM and 5 μg/mL ethidium homodimer-1. Cell proliferation was also quantified via a DNA content assay. The DNA content of the cells adhering to the hydrogel samples was determined via a Quant-iT PicoGreen dsDNA reagent kit according to the manufacturer’s protocol. The samples were rinsed three times with PBS (pH 7.4) and lysed in buffer containing 10 mM Tris (pH 8), 1 mM EDTA and 0.2% (v/v) Triton X-100. To facilitate DNA release, the samples were vortexed for 10 s every 5 min for a total of 30 min, after which they were maintained on ice. DNA fluorescence was measured at excitation and emission wavelengths of 480 and 520 nm, respectively. All of the assays were performed in triplicate.

### 2.9 Fabrication of MNs

MNs were manufactured with the formulation optimized by the experimental design and from the previously reported formulation as a bench control, where citric acid was used as a cross-linker. Two types of molds with different geometries were used to shape the materials into the desired forms. Approximately 500 mg of the formulation was dispensed into laser-engineered silicone molds, which were composed of 121 (11 × 11) conical holes, 600 μm in-depth, interspacing 300 μm and a base width of 300 μm or 196 (14 × 14) pyramidal cavities, 600 μm in-depth, interspacing 250 μm and a base width of 450 μm. Each array was centrifuged at 3500 rpm for 15 min and allowed to dry under ambient conditions for 48 h. MNs were then demolded, and the sidewalls were removed via scissors. MNs were subsequently placed in an oven (Weiss Technik UK, Loughborough, UK) at 150 °C for 20 min to facilitate cross-linking, as shown in Figure S4. However, for the control, cross-linking was performed at 130 °C for 40 min.

### 2.10 Characterization of the MNs

The MNs were fully characterized regarding the morphology, mechanical property, insertion ability, physicochemical, and biocompatibility, which were compared with the bench control used in this study.

#### 2.10.1 Morphology Examination

The morphology of the MNs was observed with a Leica EZ4W stereo optical microscope (Leica Microsystem Ltd., Milton Keynes, UK) at different magnifications so that the overall view and detailed perspective of the MNs could be captured. A TM3000 scanning electron microscope (SEM) (Hitachi Technology UK, Maidenhead, UK) was used to evaluate the surface topography of the MNs and to confirm the integrity of the needles in the patch. SEM evaluation was performed on a bevel stage under a vacuum.

#### 2.10.2 Mechanical Property Evaluation

To guarantee the ability of MN to penetrate the skin without breaking, we indirectly assessed its mechanical strength by evaluating the height reduction upon compression. Specifically, the length of the MNs before treatment was obtained via an optical microscope and recorded as *H*_0._ Then, the MAPs were attached to the cuboidal probe of the TA.XT. Plus Texture Analyzer (Stable Microsystem Ltd., Surrey, UK) with double-sided tape. The tip side of the patch was pressed against a solid surface with a consistent force. The compression was conducted in compression mode for 30 s with a force of 32 N at a downward rate of 0.5 mm/s. The length of the needles was subsequently viewed again and recorded as *H*_d_. The percentage of height reduction was determined via eq 4, and a reduction of less than 20% was deemed acceptable.
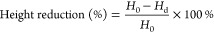
4

#### 2.10.3 Insertion Ability Assessment

The insertion of MNs was performed via Texture Analyzer and EX1301 VivoSight optical coherence tomography (OCT) (Michelson Diagnostics Ltd., Kent, UK) with a validated model of the skin and ParafilmM.^34^ The same compression procedure described in 3.9.2 was employed to conduct the insertion, and the solid surface was replaced with 8 layers of Parafilm. Upon insertion, the surface inserted with the MN was examined by an OCT, and the depth of the needle was recorded. The needles were removed, and the holes inside each layer of the Parafilm were counted under a light microscope. The percentage of holes in each layer relative to the number of MNs on the array was determined via eq 5. According to the model, the depth of each parafilm layer was approximately 126 μm, and 3–4 layers of penetration were deemed capable of penetrating the SC (10–50 μm). Layers with a penetration rate of more than 20% were considered successful. Similarly, the insertion ability of MN into porcine skin was subsequently evaluated by inserting MN into the skin for 30 s via manual thumb pressure and subsequently observed under OCT. The depth of the insertion was recorded and measured using ImageJ.
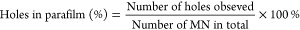
5

### 2.11 Development of the Enfuvirtide-Loaded Reservoir

#### 2.11.1 Reservoir Formulation Development

Direct compression and lyophilization were the two approaches that were preliminarily investigated for enfuvirtide reservoir fabrication in this study, which involve little or no water in the final product for the stability of enfuvirtide. For the compression method, a variety of combinations of enfuvirtide and excipients listed in Table 2 were prepared. The tablets were produced via a Manual 25Ton hydraulic press (Specac, Orpington, UK) by filling 100 mg of formulation mixture into an evacuable pellet die (10 mm) and pumping the handle until the required load was indicated on the gauge. The load was maintained for 10 s, and the tablet was ready to be removed from the die after the pressure was released, as shown in Figure S5.

**Table 2:** Formulations for the Preparation of Enfuvirtide-Loaded Compressed Tablets

		excipients		compression force (Ton)
formulation ID	enfuvirtide% w/w	mannitol% w/w	gelatin% w/w	
C1	5	85	10	2
C2	5	90	5	2
C3	5	85	10	4
C4	5	90	5	4

Lyophilized wafers for enfuvirtide were prepared using different combinations of drugs and excipients (Table 3). To prepare each formulation, gelatin, mannitol, and enfuvirtide were accurately weighed and premixed in the container. Then, deionized water was added to the mixture to make up for 100% of the weight, followed by dissolution via a speed mixer. Once a homogeneous blend was formed, the formulation was then cast into open-ended cylindrical molds with a diameter of 12.24 mm and depth of 2.5 mm, resulting in a final weight of 240 mg. Each formulation was subsequently frozen at −80 °C for 60 min and then lyophilized via a Virtis Advantage Bench Top Freeze Drier System (SP Scientific, Warminster PA, USA) with the freeze-drying cycles detailed in Table S4.

**Table 3:** Formulations for the Preparation of Enfuvirtide-Loaded Lyophilized Wafers

		excipients		water% w/w
formulation ID	enfuvirtide% w/w	mannitol% w/w	gelatin% w/w	
L1	5	20	5	70
L2	5	10	5	80
L3	5	2.5	5	87.5
L4	5	2.5	2.5	90
L5	5	2.5	1.25	91.25

#### 2.11.2 Dissolution and Hardness Tests of the Reservoirs

The dissolution time and hardness were considered two essential parameters for the reservoir in this study, as they govern the ease of release of enfuvirtide from the reservoir. The hardness of the reservoirs was evaluated via the Texture Analyzer. Specifically, the reservoir was placed on the center of the stainless-steel cubic stage and then pressed against a solid surface with a consistent force. The compression was conducted in compression mode for 30 s with a force of 32 N at a downward rate of 0.5 mm/s. The formulations that were able to withstand the force and maintain structural integrity after the compression test were considered for the following study. The dissolution time was determined via the following procedure. Initially, 25 mL of 50% ethanol was prewarmed at 37 °C and agitated via a magnetic stirring bar (6 × 25 mm) at 600 rpm. The reservoir was placed into the solution, and the time required by the reservoir to fully dissolve was recorded as the dissolution time on the basis of visual inspection. All the samples were measured in triplicate.

After the dissolution test, the resulting solution was subjected to a 100-fold dilution with 50% ethanol (v/v) before being subjected to analysis. The quantification of drug concentration (*C*) was achieved through the utilization of a meticulously developed and validated method.^32^ The recovery of enfuvirtide from each formulation was subsequently calculated by using eq 6.
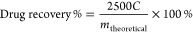
6

#### 2.11.3 Characterization of the Chosen Reservoir

The formulation exhibiting desirable dissolution time and hardness underwent additional characterization for weight and content uniformity, overall appearance, organoleptic properties, moisture content, and stability. The intra- and interbatch weights and content variation were assessed using 6 wafers from a single batch and multiple batches, respectively. Wafer dimensions were determined with a caliper. The moisture content was determined through a loss-on-drying test involving weighing, drying at 100 °C for 30 min, and reweighing. Stability was investigated by storing wafers in airtight glass containers covered with aluminum foil at ambient temperature for one month.

### 2.12 Antiviral Activity and Cytotoxicity of Enfuvirtide Lyophilized Wafers

The cell viability evaluation and virus titration procedures are presented in the Supporting Information for Publication. For the antiviral and cytotoxicity studies, stock solutions of lyophilized wafers and enfuvirtide were prepared by dissolving them in sterilized and filtered water to achieve a final enfuvirtide concentration of 1 mg/mL. Stocks for excipients gelatin and mannitol were also prepared according to their respective concentrations in the formulations. AZT was used as a positive control for HIV inhibition. The working solutions were prepared by diluting the stocks with culture medium to achieve concentrations of 150 μg/mL for enfuvirtide and 10 μg/mL for AZT. A 96-well flat-bottom sterile plate was divided into an upper set (wells 2B to 2D) for antiviral studies and a lower set (wells 2E to 2G) for cytotoxicity studies. Triplicate wells containing serial dilutions of the drugs were seeded with 30,000 MT4 cells (50 μL at 6 × 10^5^ cells/mL) and infected with 50 μL of HIV-1 NL4.3 at four times the concentration required to kill all the cells. Drug-mediated cytotoxicity was assessed in MT4 cells at a concentration of 6 × 10^5^ cells/mL, with the virus replaced with a culture medium. The last two series (11B:11D and 11E:11G) served as HIV-positive and noninfected controls, respectively.

Five days postinfection/incubation, 20 μL of MTT was added to each well. After 1 h of incubation at 37 °C in a humidified atmosphere with 5% CO_2_, 150 μL of the supernatant was carefully removed, and 150 μL of acidified Triton X-100 isopropanol solution was added to each well to allow complete dissolution of the formazan crystals overnight. Formazan production was quantified spectrophotometrically at 550/690 nm. The CC50% cytotoxic concentration (CC_50_) and 50% inhibitory concentration (IC_50_) values were calculated for each compound via nonlinear regression with a log dose vs normalized response to the data. The antiviral assay was conducted four times: initially, the appropriate starting concentration for enfuvirtide was determined, followed by three replicate experiments.

### 2.13 Ex Vivo Skin Permeation of Enfuvirtide from MNs Associated with Reservoirs

#### 2.13.1 Vertical Cell DiffusionTest

The ex vivo permeation of enfuvirtide from MNs, coupled with a reservoir, through dermatomed neonatal porcine skin (350 μm) was investigated via the modified Franz diffusion cells illustrated in Figure S6. The control group was set up by mounting the wafer on top of the skin with 20 μL of water added. The receiver compartment, which was filled with 12 mL of 50% ethanol (v/v), was stirred at 600 rpm with a 6 × 25 mm stirring bar and pre-equilibrated at 37 °C. Skin samples from stillborn piglets were carefully excised and shaved to achieve a thickness of 350 μm via an electric dermatome trimmer and stored at −20 °C. Prior to the experiment, the skin, pre-equilibrated in PBS (pH 7.4), was affixed to the donor compartment with cyanoacrylate glue. MNs were placed in the center of each donor compartment and subjected to manual pressure for 30 s. The reservoir and a 5.0 g stainless steel weight were subsequently placed on the MN. A control group was introduced where no MNs were involved. The donor compartment was mounted onto the receiver compartment and securely clamped and the sampling arm was sealed with Blu-Tack. At predetermined intervals, 200 μL of the solution was sampled and analyzed via HPLC after centrifugation.^29^

#### 2.13.2 SecondaryStructure Confirmation of Permeated Enfuvirtide

The secondary structure of enfuvirtide permeated from MNs into the release medium was evaluated via a Jasco J-815 circular dichroism (CD) spectrometer coupled with a temperature controller and nitrogen pump (Jasco, Easton, MA, United States). Samples 100 μL in volume were loaded into a high-precision quartz cuvette with a path length of 1 mm. A scan speed of 20 nm/min with five accumulations per sample was used with far-UV wavelengths ranging from 300 to 190 nm at 25 °C. The baseline was corrected by subtracting measurements of the solvent only.

#### 2.13.3 Skin Integrity Evaluation after the Permeation Study

Transepidermal water loss (TEWL) was assessed both before and after Franz cell diffusion studies to gauge the skin barrier function and macroscopic alterations. A portable evaporimeter (Delfin Technologies, Kuopio, Finland) was employed for TEWL measurement, ensuring that the closed chamber was unaffected by ambient airflow. The evaporation rate (g/m^2^ h) of the device was calculated from the increase in relative humidity (RH). Measurements were conducted before and after MN application to the skin under controlled conditions (60% humidity and 25 °C) with approximately 10 s of measurement time for each of the five measurements per skin.

### 2.14 Statistical Analysis

Statistical analysis was conducted via GraphPad Prism version 9.0 (GraphPad Software, San Diego, CA, USA). Data normality was assessed via the Shapiro–Wilk test, with a *p* value >0.05 indicating a normal distribution. For normally distributed (parametric) data, unpaired *t* tests were used for comparisons between two groups, whereas one-way and two-way analysis of variance (ANOVA) with Tukey’s post hoc tests were used for comparisons among three or more groups. The statistical significance was set at *p* < 0.05. For model selection, *p* > 0.10 indicated that models adequately describe the data without significant unexplained variability.

## 3 Results and Discussion

### 3.1 Hydrogel Swelling Properties and Enfuvirtide Diffusion Levels across Swollen Hydrogels

**Figure 3:**
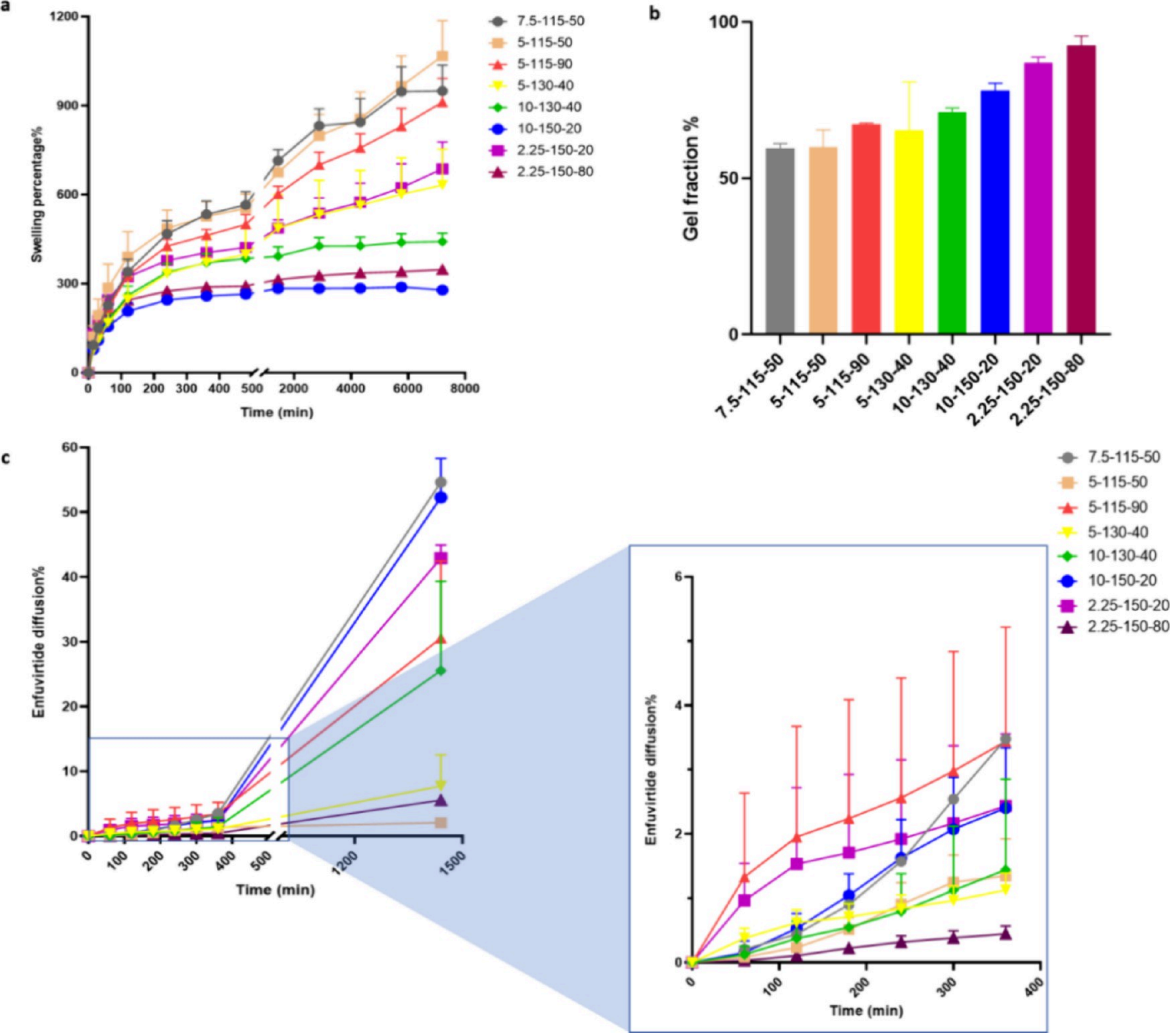
Swelling profile (a), gel fraction (b), and enfuvirtide diffusion percentage (c) of the hydrogels, denoted as the cross-linker amount (%) – temperature (°C) – time (min) (Means + SDs, *n* = 3).

The hydrogels were synthesized on the basis of software recommendations, and comprehensive studies, including swelling, gel fraction determination, diffusion tests, and rheological examinations, were systematically conducted, the results of which are presented in Figure 3. The formulations were denoted as the cross-linker ratio (% w/w)– temperature (°C) – time (min).

A notable disparity in swelling behavior was detected between formulations 10–150–20 and 2.25–150–80 relative to the other formulations from 24 h onward, a pattern that remained consistent over the subsequent 5 days (*p* < 0.05). Moreover, formulations 10–150–20, 2.25–150–20, and 2.25–150–80 exhibited significantly greater gel fractions than the rest (*p* < 0.05). In terms of drug diffusion, formulations 5–130–40, 2.25–150–80, and 5–115–50 demonstrated substantially lower permeation rates over a 24 h period compared to other formulations (*p* < 0.05). The decrease in swelling, as observed in Figure 3a, correlated with an increase in the cross-linking temperature, time, and cross-linker amount. This trend suggested an increase in ester bond formation. The increased temperature and prolonged heat duration facilitated more reactions by increasing the collision frequency and energy, thereby promoting effective collisions. Additionally, the elevated temperature expedited the removal of water as a reaction product, driving the esterification process to the right. The increased amount of cross-linker, following Le Chatelier’s principle, favored the esterification equilibrium toward the formation of more products.^35^ According to Figure 3b, the gel fraction significantly increased with increasing cross-linking time and temperature for formulations 2.25–150–80 compared with the other formulations (*p* < 0.05), indicating a pronounced influence on the thermal treatment parameters. After 24 h of swelling, most of the hydrogels nearly reached equilibrium, and the diffusion of energy was governed by the osmotic pressure, enfuvirtide-hydrogel interaction, and hydrogel properties, such as the mesh size, thickness, and density. Fick’s law of diffusion, expressed as eq 7 with *J* as the flux, *D* as the diffusion coefficient, and dφ/d*x* as the concentration gradient, governed enfuvirtide diffusion through the hydrogel.^36^ Distinct hydrogel characteristics, such as a larger mesh size and lower density, facilitated faster movement of enfuvirtide, and thicker hydrogels impeded diffusion by influencing the parameters dφ/d*x*. Additionally, interactions, such as hydrogen bonding or electrostatic interactions, affect the effective diffusion coefficient. Notably, except for formulations 5–115–50 and 2.25–150–80, there were no significant differences in the 24 h diffusion of enfuvirtide through the hydrogels observed in this study (Figure 3c).
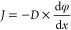
7

**Figure 4:**
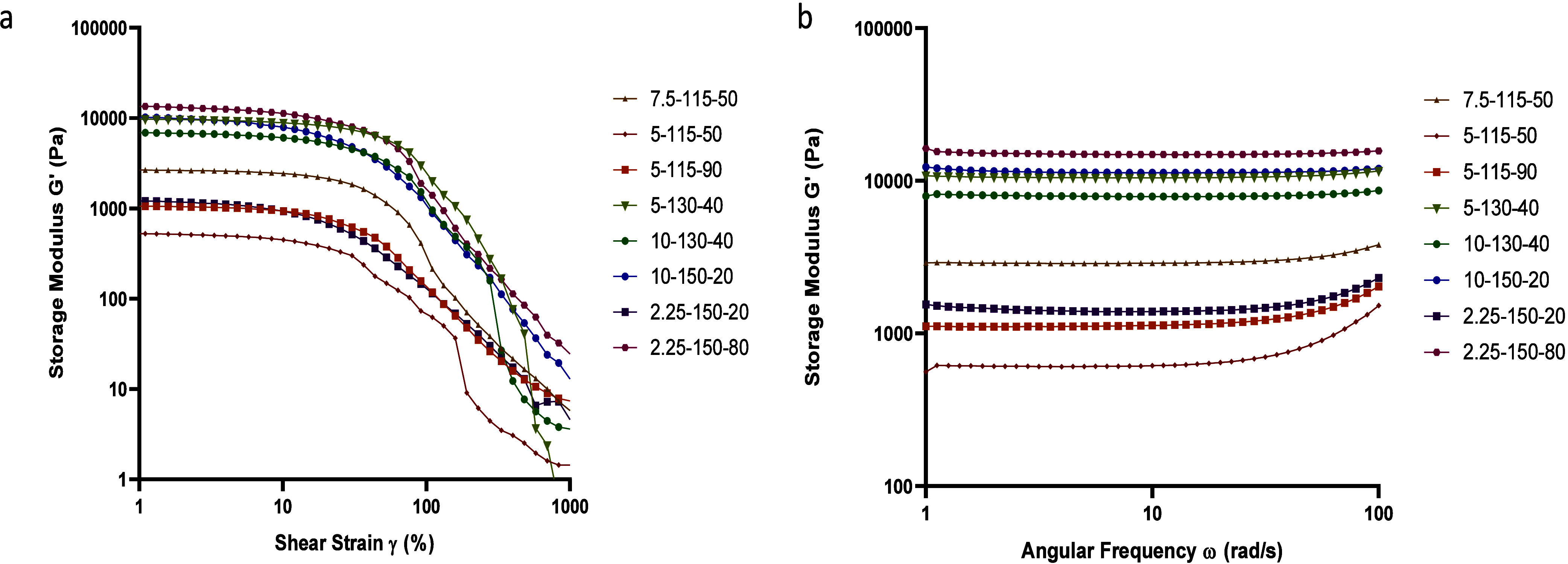
Storage modulus of hydrogels from amplitude sweeps (a) and frequency sweeps (b), denoted as the cross-linker amount (%) – temperature (°C) – time (min).

Amplitude sweeps, conducted alongside controlled shear deformation, elucidated the crucial rheological characteristics of the hydrogels. Parameters such as the linear viscoelastic region (LVER), yield point (τ_y_), and flow point (τ_f_) were extracted from the shear strain plot (Figure 4a). The LVER limit, determined by the software with a 5% *G*′ deviation tolerance around the plateau value, indicated the maximum strain where the hydrogel structure remained intact. The yield point (τ_y_) represents the shear stress at the LVER limit, whereas the flow point (τ_f_) represents the shear stress at the crossover of the storage modulus (*G*′) and loss modulus (*G*″) (data not shown). In the yield zone between the yield point and flow point, where *G*′ > *G*″, the initial structural strength of the hydrogel decreased, but the hydrogel predominantly maintained solid behavior.^37^

Table 4 summarizes the LVER limit, proposed LVER, and flow point of the hydrogels. The LVER for all formulations was achieved beyond the expected LVER threshold, validating the accuracy of the test. In samples cross-linked at 115 °C, increasing the cross-linker concentration from 5 to 7.5% had a more pronounced effect on gel strength than extending the cross-linking duration from 50 to 90 min, as shown by a storage modulus (*G*′) of 2580 Pa for the 7.5–115–50 formulation compared to 955 Pa for the 5–115–90 formulation. This trend was consistent in hydrogels cross-linked at 150 °C, where a formulation with a 10% cross-linker achieved a *G*′ of 10,220 Pa, in contrast to 1200 Pa in a 2.25% cross-linker formulation, both processed for 20 min. Additionally, all formulations demonstrated relatively high strain and stress at the flow point, with shear strain values exceeding 50%, indicating that the hydrogel could withstand substantial deformation before irreversible structural damage. This property is advantageous for hydrogel-forming MN applications, where intact removal after drug delivery is required. Notably, formulation 5–115–50, with the lowest storage modulus, presented the lowest drug diffusion percentage. Conversely, formulation 2.25–150–80, which had the highest gel strength, demonstrated the second-lowest enfuvirtide diffusion. This association highlights the crucial role of gel strength in regulating molecular diffusion within the matrix. Extreme gel strength values, whether excessively high or low, yielded comparably less molecular diffusion. Elevated gel strength, linked to a dense structure and smaller pores, impedes molecular movement and decelerates diffusion. Conversely, low gel strength results in structural instability, obstructing molecular movement. Striking the optimal balance in gel strength is imperative for attaining the desired diffusion characteristics, particularly in applications such as drug delivery systems and biomaterials. This study also indicated that gel strength could serve as a potential critical quality attribute instead of drug diffusion, though this depends on the drug’s physicochemical properties and potential interactions with the hydrogel. The frequency sweep results, which complemented the amplitude sweeps, confirmed the stability of the hydrogel by demonstrating a consistent *G*′ with increasing angular oscillatory frequency (ω) (Figure 4b) and well-separated yield points and flow points (Table 4).

**Table 4:** LVER Limit, LVER Proposal, and Flow Point of the Hydrogels

	LVER limit		LVER proposal		flow point		
hydrogel	strain (%)	*G*′ (Pa)	strain (%)	stress (Pa)	stress (Pa)	strain (%)	*G*′ (Pa)
7.5–115–50	2.9	2580	1	26.6	643.3	81.37	582.9
5–115–50	1.43	520	1	5.27	96.24	90.61	75.2
5–115–90	8.56	955	5	50.3	242	68.58	250.7
5–130–40	2.93	9500	1	97.5	3286	106.3	2194
10–130–40	1.98	6770	1	69	2022	89.88	1595
10–150–20	0.929	10,220	0.5	52.3	1931	71.68	1906
2.25–150–20	1.24	1200	0.5	6.19	212.6	54.28	277.1
2.25–150–80	0.979	13,500	0.5	69.1	2729	87.34	2222

**Figure 5:**
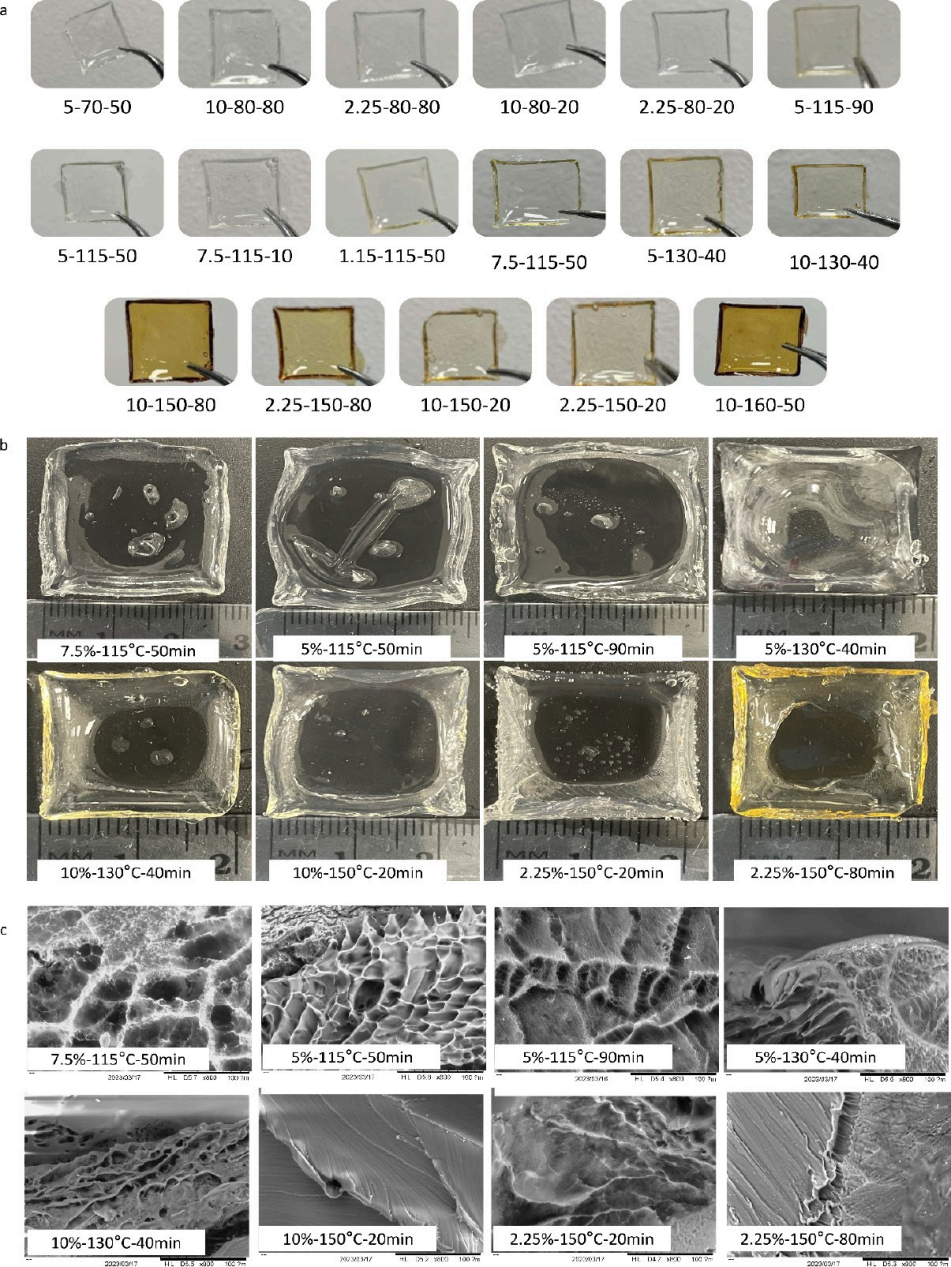
Morphology of the hydrogels before (a) and after swelling (b), micromorphology, (c) after swelling, denoted as the cross-linker amount (%) – temperature (°C) – time (min).

The macro- and micromorphologies of the hydrogel films before and after swelling are depicted in Figure 5. With the exception of hydrogels cross-linked at 70 and 80 °C and formulations 5–115–50 and 7.5–115–10, the remaining films exhibited a noticeable color transformation from transparent to yellowish after undergoing specific thermal treatments. Notably, formulations 10–150–80 and 10–160–50 presented a distinct dark brown hue (Figure 5a). The color shift observed in hydrogel formulations following thermal treatment may result from the formation of new chemical groups that absorb light and produce a complementary color. However, the newly formed groups responsible for the visible color changes could not be confirmed by infrared spectroscopy in this study, likely due to their low concentrations falling below the detection limit, one of the study’s limitations. Despite being undetectable via this method, similar color changes have been reported in previous studies, likely due to structural modifications in PVA. Specifically, these studies noted the emergence of a strong band around 1700 cm^–1^ in FTIR spectra, which is only weakly present in the initial PVA. This band is hypothesized to result from polymer degradation or oxidation, correlating with the observed color changes.^38^ The in vivo biological safety of the PVA-based hydrogels despite the color change was demonstrated by the consistent average body weight of the mice throughout the study, with no observable adverse effects or toxicities detected in various organs upon histopathology examination.^39^ The 6 films shown in Figure 5b with an appropriate elastic property and an intact surface after swelling were tested via diffusion studies. SEM images of the cross-sectional micromorphology of the hydrogels revealed distinct variations in the pore size (Figure 5c). Hydrogels cross-linked under relatively mild conditions, such as formulations 7.5–115–50, 5–115–50, 5–115–90, 5–130–40, 10–130–40, and 2.25–150–20, exhibited a well-organized network of interconnected pores with uniformly distributed pore sizes. The surface displayed homogeneity, featuring a well-defined porous texture without any noticeable signs of cracks or irregularities. In contrast, formulations 10–150–20 and 2.25–150–80 revealed a finer and more densely packed network with smaller pores. These variations in microarchitecture underscore the diverse structural characteristics and porosity evident among the hydrogel formulations.

### 3.2 Response Surface Methodology for MN Optimization

**Figure 6:**
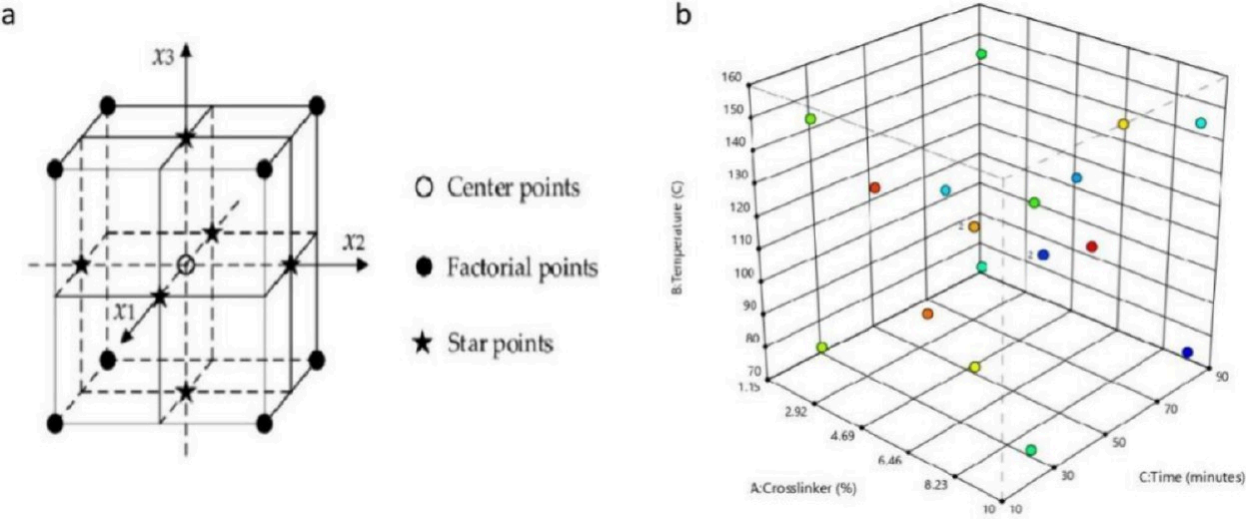
Three-factor layout for the centered central composite design (a) and the scatter plot of the design of this study (b).

The central composite design was chosen in this study as a factorial design with center points augmented with a group of axial points, which allowed for the curvature to be estimated (Figure 6a). The 2-level factorial points were used to estimate the linear effects and two-factor interactions. The center points were used to estimate quadratic effects and were replicated to estimate the pure error of the experiments and help tie the blocks together. Axial (star) points were used to estimate pure quadratic effects.^40^ The positions of the modeling points are shown in Figure 6b.

**Figure 7:**
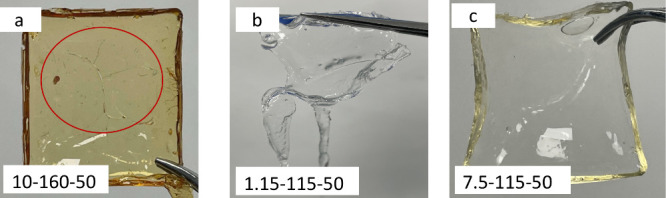
Examples of extremely rigid (a), weak (b), and normal (c) swollen hydrogel films, denoted as the cross-linker amount (%) – temperature (°C) – time (min). Red circle in (a) highlights the crack of this formulation after swelling.

The experimental response data were derived from a series of experiments recommended by the software, and the observations are systematically documented in Table 5. Instances where hydrogel formation was unsuccessful or resulted in excessively brittle (Figure 7a) and viscous hydrogels (Figure 7b), were excluded from model fitting to avoid confusion in the analysis. Figure 7c exemplifies the normal hydrogel used for diffusion studies. Swelling in 24 h (Swelling_24h_) served as the response variable instead of the EWC. This substitution was made because some hydrogels continued to exhibit increased swelling even after 5 days, making the swelling percentage in the initial 24 h a more practical and standardized measure.

**Table 5:** Results of Experimental Trials for PVA/PVP/PEGdiacid–Based Hydrogel Formulations

	factors			Rponses		
run	cross-linker% w/w	temperature °C	time (min)	swelling_24h_% w/w	GF% w/w	diffusion% w/w
1	10	80	80	NA	NA	NA
2	7.5	115	50	707.6	50.2	50.77
3	7.5	115	50	714.2	49.66	54.61
4	5	115	90	601.92	57.26	30.58
5	5	130	40	486.12	66.37	7.72
6	10	150	80	114.32	90.12	NA
7	2.25	80	80	NA	NA	NA
8	10	80	20	NA	NA	NA
9	2.25	150	80	313.49	82.95	5.56
10	5	115	50	675.01	40.85	2.05
11	10	150	20	283.60	69.62	52.29
12	2.25	150	20	487.22	64.78	42.92
13	2.25	80	20	NA	NA	NA
14	5	70	50	NA	NA	NA
15	10	160	50	159.11	88.41	NA
16	5	115	50	675.01	40.85	2.05
17	7.5	115	10	NA	NA	NA
18	1.15	115	50	552.35	44.94	NA
19	10	130	40	392.18	66.37	25.56

The initial phase of mathematical modeling involved fitting the experimental data to an appropriate model, guided by parameters such as the sequential *p* value, lack of fit *p* value, adjusted *R*^2^, and predicted residual sum of squares (PRESS) values obtained from regression analysis. Subsequently, ANOVA was applied to assess the significance of the model at the 5% significance level. In cases where more than one model demonstrated significance (*p* < 0.05) for a given response, the adjusted *R*^2^ and PRESS values of each model were scrutinized to identify the most suitable mathematical model. The sequential *p* value was expected to be less than 0.05, whereas the lack of fit *p* value was intended to exceed 0.10, guided by the Design Expert.^41^ The primary focus was on maximizing the adjusted *R*^2^ and predicted *R*^2^, and lowering the PRESS value, indicating adequate model fitting.^42−44^

**Figure 8:**
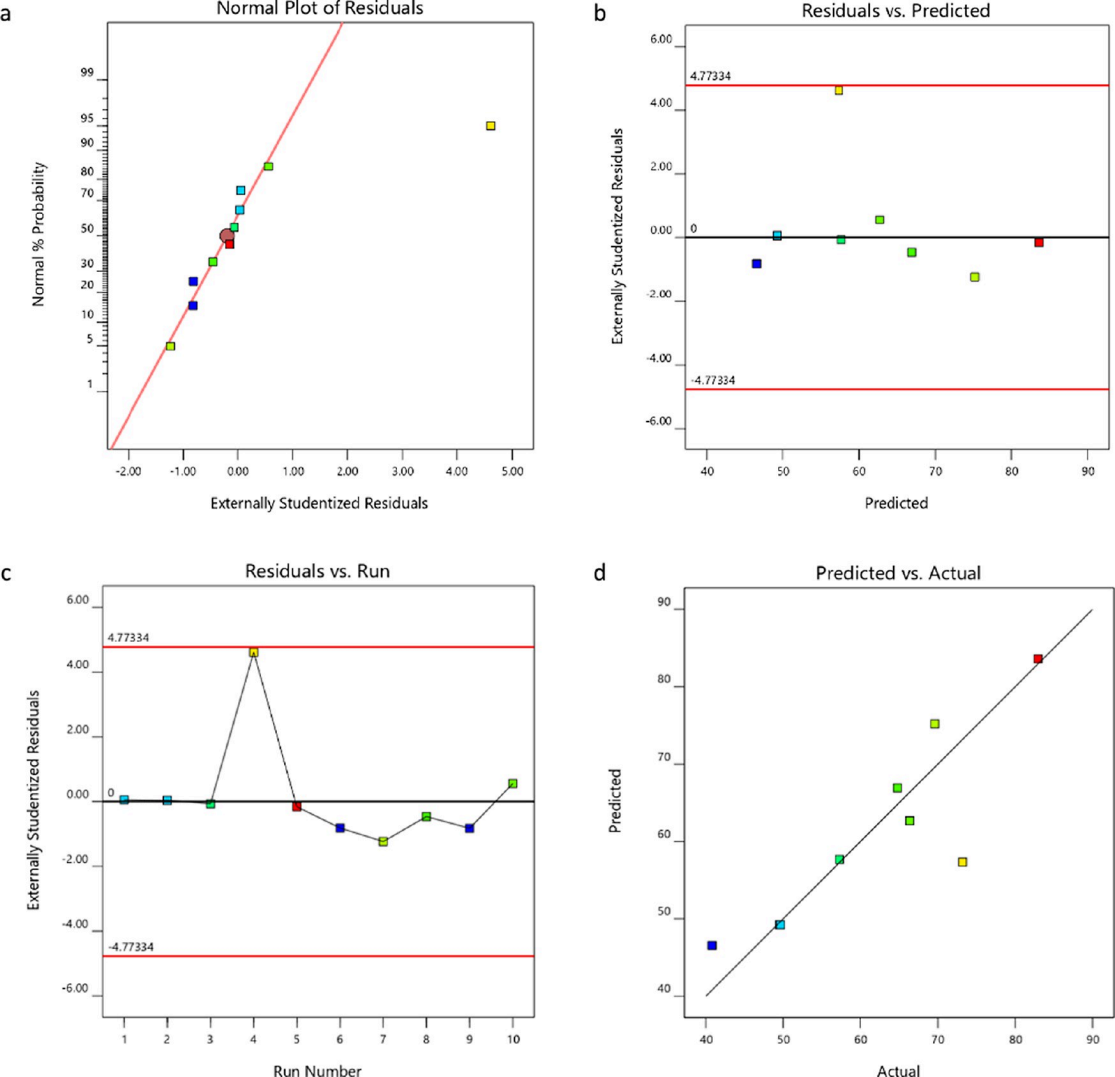
A diagnostic of the model for gel fraction. (a) Normal plot of the residuals, (b) residuals vs predicted, (c) residuals vs run plot, and (d) predicted vs actual plot.

Table 6 presents a fit summary of the suggested models for the three responses, whereas Figure 8 illustrates a diagnostic example for the gel fraction model. Specifically, the chosen models for Swelling_24h_, gel fraction, and diffusion were two-factor interaction (2FI), linear, and 2FI, respectively, which presented desirable *p* and *R*^2^ values. Preference was given to models where the difference between the adjusted *R*^2^ and the predicted *R*^2^ was less than 0.2. Adeq Precision, which measures the signal-to-noise ratio, indicates ratios greater than 4 for all the models, indicating an adequate signal and supporting the models’ use in navigating the design space. For the validation of the ANOVA, four diagnostic plots were examined. In the case of the gel fraction linear model, the normal plot of the residuals displayed a fairly straight line (Figure 8a), as preferred. The residuals vs predicted and residuals vs run plots were anticipated to exhibit no discernible patterns (Figure 8b,c). The Predicted vs Actual plot, depicted in Figure 8d, indicated an even distribution of actual values on both sides of the predicted value line. These diagnostics were also conducted for the Swelling_24h_ and diffusion models.

**Table 6:** Fit Summary and Fit Statistics of the Suggested Models for Three Responses

response	source	sequential *p* value	lack of fit *p* value	adjusted *R*^2^	press	adeq precision
swelling_24h_	2FI	<0.0001	0.8574	0.9535	20,660	94.6643
GF	Linear	0.0168	0.4836	0.9408	1251.86	18.2209
diffusion	2FI	0.0029	0.1442	0.9704	62,526.10	23.2611

**Figure 9:**
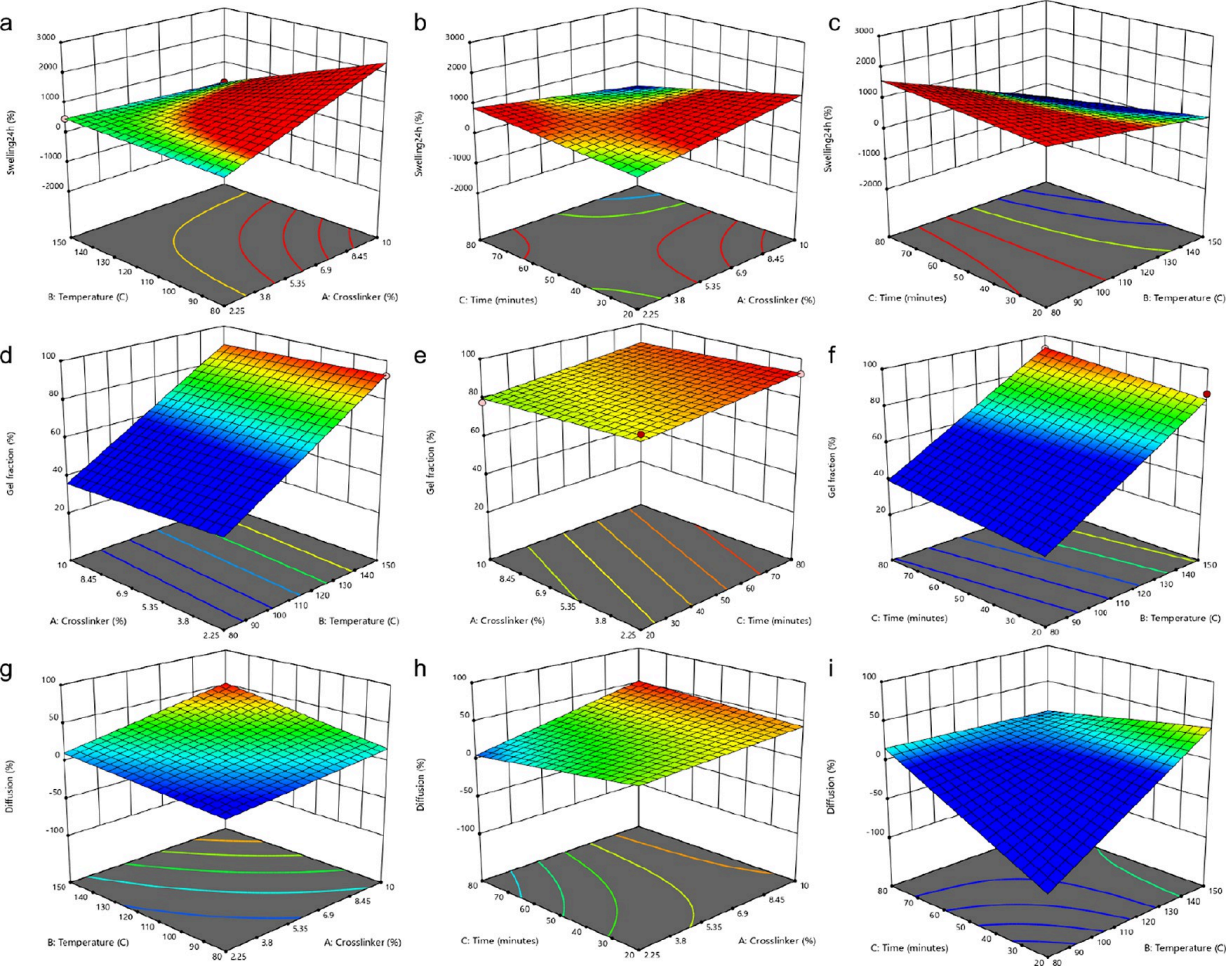
Response surface plots showing the effects of various variables on the 24-h swelling percentage (a–c) and gel fraction (d–f) and on energy diffusion across hydrogels (g–i), denoted as the cross-linker amount (%) – temperature (°C) – time (min).

Mathematical relationships between factors and responses were generated through response surface regression analysis via Design-Expert 7.0 software, where polynomial models, incorporating interaction and quadratic terms (if applicable), were created, as demonstrated in eq 8 as an example.^40,45^ In the model, β0 served as the intercept, representing the arithmetic averages of all of the quantitative outcomes from the 10 runs. Coefficients β1 to β5 were derived from the observed experimental values of *Y*. Variables *X*1 and *X*2 denoted the coded levels of factors. The terms *X*1 × 2, *X*1 × 3 and *X*2 × 3 and *Xi*^2^ (*i* ϵ {1, 2, 3}) represent the interaction and quadratic terms, respectively. Coefficients involving a single factor indicated the effect of that specific factor, whereas those involving multiple factors and second-order terms signified the interaction between those factors and the quadratic nature of the phenomena, respectively. A positive sign indicates a synergistic effect, whereas a negative sign denotes an antagonistic effect of the factor.^46^ Three-dimensional (3D) response surface graphs illustrating the impacts of the most statistically significant variables on the evaluated parameters are presented in Figure 9.
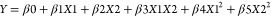
8

From the fit summary, the linear model and 2FI model were found to be significant for Swelling_24h_. The 2FI model was selected on the basis of the maximum value of Adjusted *R*^2^ and low PRESS value, indicating an adequate fit of the model. The 2FI equation generated by the software is shown in eq 9, where *Y*, swelling_24h_, *X*1, the ratio of PEGdiacid, *X*2, the cross-linking temperature, and *X*3, the cross-linking time.
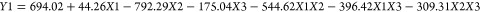
9

Equation 9 demonstrates the significant impacts of all three factors on the swelling characteristics of the hydrogel. Specifically, an increase in the ratio of PEGdiacid had a positive effect, whereas both the cross-linking temperature and time had negative effects. Notably, the change in temperature had a more pronounced effect compared to the other two factors. The combined effect of factors *X*1, *X*2, and *X*3 was revealed by the coefficients in front of the terms and could be further elucidated with the help of response surface plots. Figure 9a–c show that swelling varied in a linear fashion with the amount of cross-linker, cross-linking time, and temperature. In the context of varying cross-linkers and temperatures, the role of the cross-linker predominated, as evidenced by a steeper ascent on its side of the response surface. This could be attributed to the pore-forming effect of excessive acid, which dissolved more rapidly posthydrogel swelling. The presence of excess acid was confirmed through the pH alteration of deionized water after swelling. Additionally, solvent molecules may permeate the hydrogel due to hydrogen bond interactions with carboxylic acids at both ends of PEGdiacid, and electrostatic repulsion between the negatively charged carboxyl groups causes uncoiling and expansion of molecules, leading to increased swelling. Nonetheless, comparable significance was observed in the response surface plots for the cross-linker and time-influencing swelling at a fixed temperature of 115 °C. The swelling percentage consistently remained high when cross-linking occurred at lower temperatures for extended durations, indicating restrained ester bond formation. This phenomenon could be attributed to the requirement for specific energy activation in esterification, as dictated by the Arrhenius Equation.^47^

A linear model was suggested to fit the gel fraction with an equation generated by the software shown in eq 10, where *Y*, gel fraction, *X*1, ratio of PEGdiacid, *X*2, cross-linking temperature, and *X*3, cross-linking time. Three factors significantly affect the gel fraction, with an antagonistic effect from the cross-linker amount and a synergistic effect from increased thermal treatment, governed by the cross-linking temperature and time. Notably, no apparent two-factor interactions were observed in the surface plot depicted in Figure 9d–f.
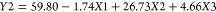
10

The optimized variables fit the first-order polynomial equation of enfuvirtide diffusion percentage well, as shown in eq 11, where *Y*, diffusion percentage, *X*1, ratio of PEGdiacid, *X*2, cross-linking temperature, and *X*3, cross-linking time.
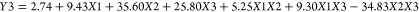
11

All three factors individually had a positive impact on the diffusion percentage when examined in an appropriate range, with a significant sequence cross-linking temperature > cross-linking time > ratio of PEGdiacid. The combination of changes in the amount of cross-linker with both temperature and time has a positive effect on the diffusion level. However, the simultaneous increase in both cross-linking time and temperature significantly hindered drug permeation through the microstructure of the hydrogel in a nonlinear manner. This could be attributed to higher temperatures providing additional thermal energy to the polymer chains, resulting in increased molecular motion and agitation. This heightened movement facilitated the formation of cross-links, contributing to a more compact and closely knit hydrogel structure with reduced chain mobility, as confirmed by the micromorphology. Moreover, elevated temperatures accelerated solvent evaporation from the hydrogel matrix. As the water evaporated, the polymer chains came closer together, forming a tighter network with smaller pores. This process also aggregated hydrophobic domains and minimized the hydrophobic surface area in contact with the bulk water, thus decreasing microchannel formation.

When cross-linked at relatively low temperatures, hydrogels become progressively rubbery because of the uncoiling of polymer chains and the subsequent increased mobility of the polymer chains after swelling. It absorbs a significant amount of water and ultimately loses its ability to retain shape. This outcome was undesirable for the production of MNs as the viscosity of the gel layer increased, thus limiting the release of the active ingredient. The surface response plots for the percentage of diffusion in Figure 9g–i exhibited a negative result in the modeling, which is unlikely to occur in actual experiments. This discrepancy may be attributed to the limited number of runs in the diffusion study, as some hydrogel samples were too weak or too rigid and cracked after swelling. This resulted in inadequate accuracy of model predictions.

After conducting thorough feasibility assessments and exhaustive grid searches across the entire experimental domain, a formulation cast from aqueous blends containing 2.25% w/w PEGdiacid, 10% w/w PVP, and 15% w/w PVA, cross-linked at 150 °C for 20 min, demonstrated the highest desirability of 0.871 and was selected for MN fabrication. The chosen optimal formulation, identified as checkpoints, underwent preparation and evaluation to validate the experimental design and polynomial equations. The experimental data for the response properties were subsequently quantitatively compared with the predicted values. The formulation exhibited a swelling percentage of 490.72% ± 3.2%, a gel fraction of 75.06% ± 2.85%, and an energy diffusion percentage of 41.33 ± 5.4% in the confirmation runs (*n* = 4), where the mean of each response fell within the 95% prediction interval. The acceptable agreement between the predicted and experimental values confirmed the reliability of the optimization model used.

### 3.3 Physicochemical Property of Hydrogels

**Figure 10:**
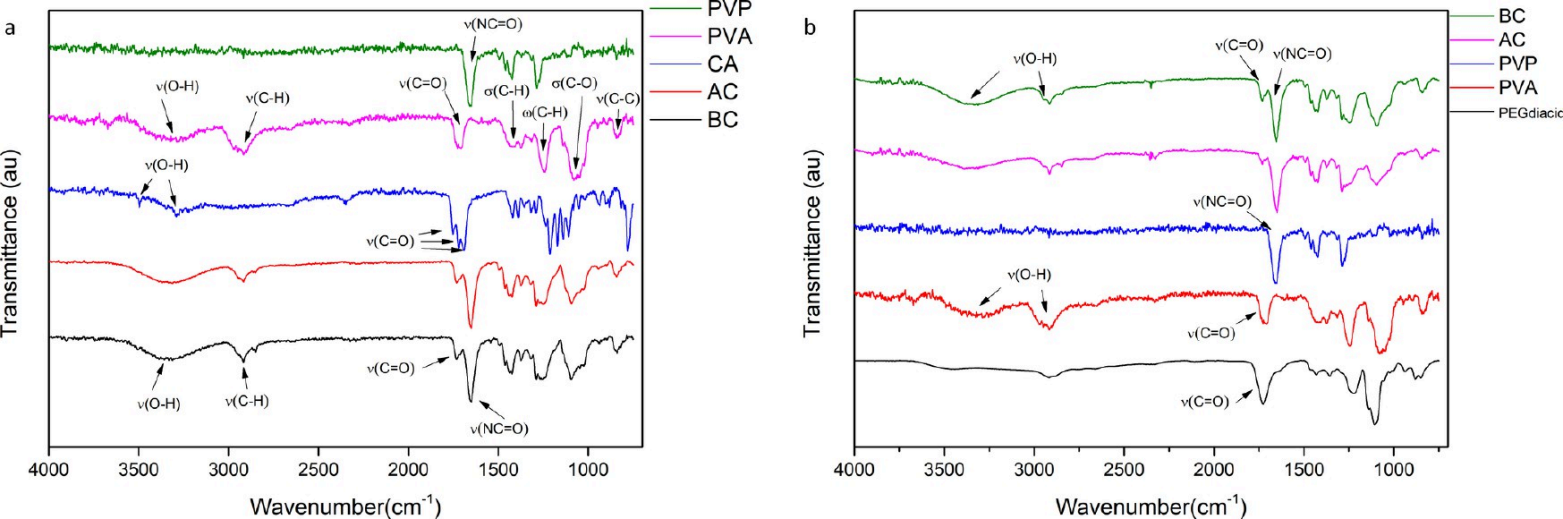
FTIR spectra of the F1 (a) and F2 (b) hydrogels. F1: cast from aqueous blend containing citric acid (1.5% w/w), PVP (10% w/w), and PVA (15% w/w) cross-linked at 130 °C for 40 min. F2: cast from aqueous blend containing PEGdiacid (2.25% w/w), PVP (10% w/w), and PVA (15% w/w) cross-linked at 150 °C for 20 min.

FTIR analysis revealed chemical shifts in the formulations of PVA/PVP/citric acid (F1) and PVA/PVP/PEGdiacid (F2) after heat treatment. The IR spectra of the hydrogels and their compositions are shown in Figure 10, with interesting parts highlighted in Table 7. PVP exhibited a sharp peak at 1654 cm^–1^, which was attributed to the NC=O stretching in its pyrrolidone ring.^48^ The C=O stretch for citric acid was split into three, which was in line with a previous report.^48^ Unlike the broad and distinct stretching vibrations at 3295 and 2914 cm^–1^ produced by the hydroxyl group in PVA, the −OH in the carboxyl group in citric acid and PEGdiacid exhibited two sharp and narrow peaks at 3492 and 3285 cm^–1^. The PVA used in this study was 87–89% hydrolyzed; therefore, the C=O stretch at 1713–1732 cm^–1^ from the ester group would exist in the spectrum before cross-linking, so that even if the hydroxyl group of PVA and the carboxyl group of the cross-linker underwent an esterification reaction, the peak shift would not be reflected. To demonstrate that the chemical reaction occurred during heating, swelling studies of control hydrogels made with each pure material and material in pairs were carried out. These hydrogels quickly dissolved in PBS and water, confirming the process of cross-linking between the polymers in the formulation to some extent.

**Table 7:** Vibrational Modes of Interest in the Dried Hydrogel and Its Constituents^a^

	wavenumber (cm^–1^)							
vibrational modes	PVA	PVP	citric acid	PEGdiacid	F1	F1c	F2	F2c
ν(O–H)	3295		34,923,285	3457	3334	3318	3350	3350
ν(C–H)	2914			2919	2910	2915	2912	2919
ν(C=O)	1728		175,817,151,685	1728	1733	1729	1740	1728
ν(NC=O)		1654			1645	1652	1659	1647
(C–N)		1418			1422	1426	1430	1424
δ(C–H)	1418	1266			1276	1260	1246	1287

a F1, cross-linked with citric acid; F2, crosslinked with PEGdiacid.

**Figure 11:**
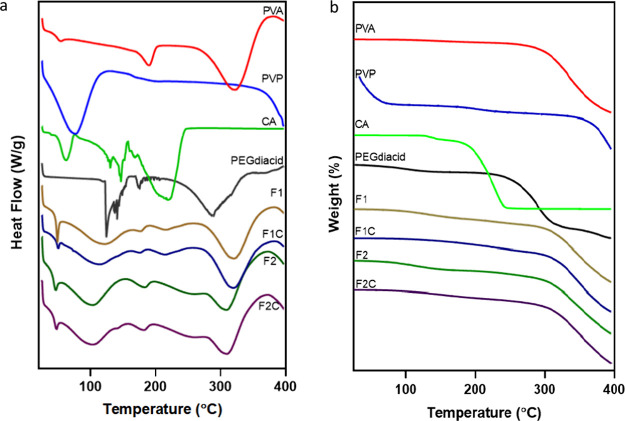
DSC (a) and TGA (b) thermograms of the hydrogel compositions before and after cross-linking. F1, cast from aqueous blend containing citric acid (1.5% w/w), PVP (10% w/w) and PVA (15% w/w) cross-linked at 130 °C for 40 min. F2, cast from aqueous blend containing PEGdiacid (2.25% w/w), PVP (10% w/w) and PVA (15% w/w) cross-linked at 150 °C for 20 min.

The thermograms of the hydrogels and their individual compositions are shown in Figure 11, and the thermal events are summarized in Table 8. The glass transition temperature (*T*_g_) of PVA was determined to be 48.04 °C via software, with an onset temperature of 44.47 °C and an end point temperature of 51.77 °C. Owing to the enthalpy relaxation, a small heat absorption peak after the *T*_g_ was observed.^49^ The melting point (*T*_m_) of PVA was indicated by a small, relatively sharp peak with a maximum of 202.22 °C, indicating its crystalline property. The significant endothermic peak on the right-hand side, within the temperature range of 270–370 °C, represented the thermal pyrolysis of PVA, with a heat consumption of 398.9 J/g and a maximum weight of 60%. DSC thermograms of PVP revealed glass transition temperatures at approximately 169.09 °C, with the absence of a melting peak (Figure 11a), which suggested the amorphous characteristics of PVP.^50^ In addition, a broad endothermic peak ranging from 28 to 127 °C was observed in the thermogram of PVP, which was attributed to water loss from the hygroscopic polymers upon heating. The citric acid used in this study was monohydrate, so the first endothermic peak at 62.22 °C corresponded to the desolvation of H_2_O in the citric acid monohydrate. As a crystallized material, a distinct endothermic peak for melting was observed from the citric acid DSC thermogram, along with an obvious weight loss from TGA (Figure 11b). Additionally, at temperatures less than 250 °C, citric acid completely decomposes (as confirmed by 100% weight loss in the TGA thermogram).^51^ The thermogram of PEGdiacid revealed decomposition of the carboxyl group endotherm with an onset temperature of 124.04 °C and decomposition of the C–C bond cleavage endotherm from 212 to 345 °C. Desolvation peaks existed both before and after cross-linking in F1 and F2, which may have been caused by the existence of hygroscopic PVP in the three formulations, which were stored for different lengths of time. For all the formulations, broad glass transition peaks were observed between 55 °C ∼ 163 °C, indicating that an amorphous hydrogel formed after heating. The peak attributed to the decomposition of citric acid at approximately 217.97 °C still existed in F1 before and after cross-linking, which may be due to the incomplete reaction of citric acid or the formation of different cross-linked structures by citric acid, resulting in some groups not participating in the reaction. No obvious peak shift was revealed by the thermogram of the hydrogel before and after cross-linking.

**Table 8:** Thermal Events of the Corresponding Peaks and Weight Loss of Peaks F1 and F2^a^

	PVA		PVP		citric acid		PEGdiacid		F1		F1c		F2		F2c	
thermal event	temp (°C)	WL (%)	temp (°C)	WL (%)	temp (°C)	WL (%)	temp (°C)	WL (%)	temp (°C)	WL (%)	temp (°C)	WL (%)	temp (°C)	WL (%)	temp (°C)	WL (%)
glass-transition	48.04	0.05	169.09	8.59					48.8	0.02	50.24	0.07	45.45	0.28	47.37	0.24
desolvation			73.24	7.79	62.22	0.19										
melting	190.18	2.46			147.52	5.48			121.17	2.84	112.54	0.71	102.96	2.24	102.96	2.24
decomposition					217.97	51.94	124.04	9	217.06	6.38	218.00	2.82	181.07	5.83	182.99	5.87
C–C bond cleavage	323.74	26.72					286.98	53.17	319.09	17.16	319.57	14.34	308.07	12.57	308.07	21.69

a F1, cast from an aqueous blend containing citric acid (1.5% w/w), PVP (10% w/w) and PVA (15% w/w) crosslinked at 130 °C for 40 min. F2, cast from aqueous blend containing PEGdiacid (2.25% w/w), PVP (10% w/w) and PVA (15% w/w) cross-linked at 150 °C for 20 min.

**Figure 12:**
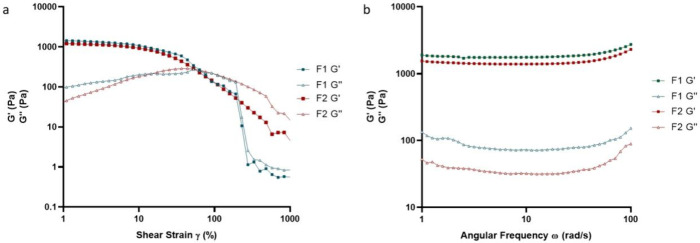
Amplitude sweeps (a) and frequency sweeps (b) of the F1 and F2 hydrogels. F1, cast from aqueous blend containing citric acid (1.5% w/w), PVP (10% w/w) and PVA (15% w/w) cross-linked at 130 °C for 40 min. F2, cast from aqueous blend containing PEGdiacid (2.25% w/w), PVP (10% w/w) and PVA (15% w/w) cross-linked at 150 °C for 20 min.

The oscillatory rheology results of the hydrogels made from F1 and F2 are presented in Figure 12. The amplitude sweeps revealed a synchronous variation in the storage modulus (*G*′) plots, demonstrating comparable yields and flow points. These plots intersected with the loss modulus (*G*″) plot at the corresponding points. Notably, a substantial decrease in *G*′ was observed for F1 after 100% deformation of the hydrogel, indicating the occurrence of brittle fracture, a phenomenon absent in F2 (Figure 12a). The frequency sweeps depicted a consistent *G*′ for both hydrogels, with increased angular oscillatory frequency (Figure 12b), indicating desirable stability.^52^ This observation reinforced the robustness and reliability of the hydrogels under the applied conditions.

### 3.4 Biocompatibility of the Hydrogels

**Figure 13:**
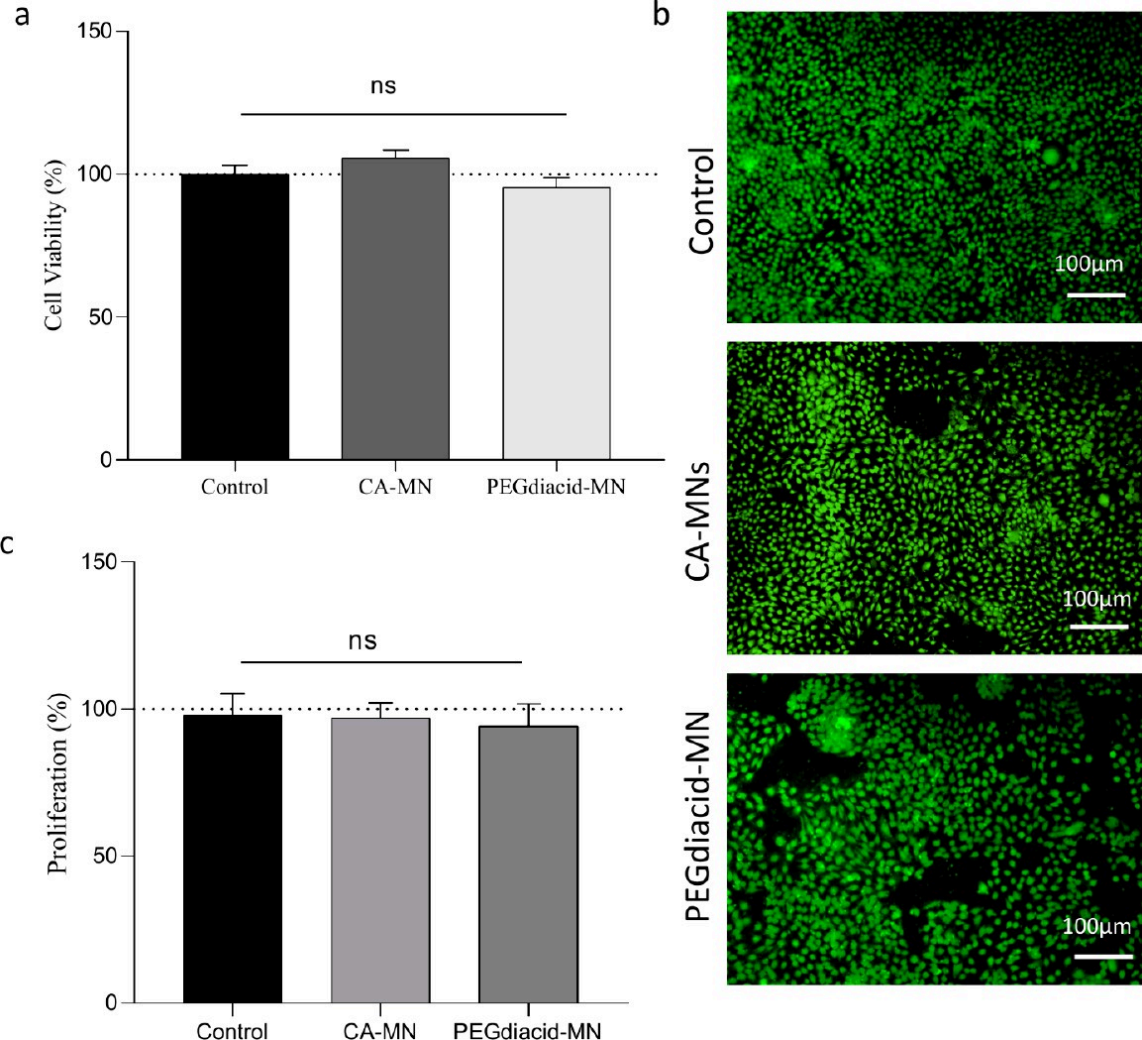
(a) Cell viability (%) after 72 h of exposure to CA-MN and PEGdiacid-MN in the MTT assay. (b) Live/dead staining of human keratinocytes treated with control, CA-MN or PEGdiacid-MN (green: live; red: dead). (c) Total DNA content in the control, CA-MN, and PEGdiacid-MN samples after 72 h, as measured by the PicoGreen assay (Means + SD, *n* = 6).

In this study, the MTT assay was employed to assess the potential cytotoxic effects of the hydrogels on human keratinocytes. As shown in Figure 13, the percentages of viable CA-MN (PVA/PVP/citric acid–based hydrogel) and PEG-MN (PVA/PVP/PEGdiacid–based hydrogel)-treated cells were 105.6 and 95.4%, respectively, after 72 h of exposure. Statistical analysis revealed no significant differences between the control group and either formulation, with cytotoxicity levels recorded as 0 and 1, respectively. According to the ISO 10993–5 standards, this confirms the absence of toxicity.^53^ Furthermore, calcein/ethidium homodimer-1 staining revealed no red fluorescence in cells treated with CA-MN or PEG-MN, indicating minimal extracellular nucleic acid release from dead cells with compromised plasma membranes (Figure 13b). These observations, along with the results shown in Figure 13a, strongly support the conclusion that the CA-MN and PEG-MN formulations did not compromise the viability or plasma membrane integrity of the keratinocyte cells. Additionally, a PicoGreen assay was conducted to evaluate the impact of the formulations on cell proliferation over a 72 h period. The data revealed that neither CA-MN nor PEG-MN negatively affected cell proliferation (Figure 13c). Overall, the findings confirm that both formulations are noncytotoxic to keratinocytes, suggesting that CA-MN and PEG-MN could be safe for human skin applications.

### 3.5 Morphology and Geometry of the MNs

**Figure 14:**
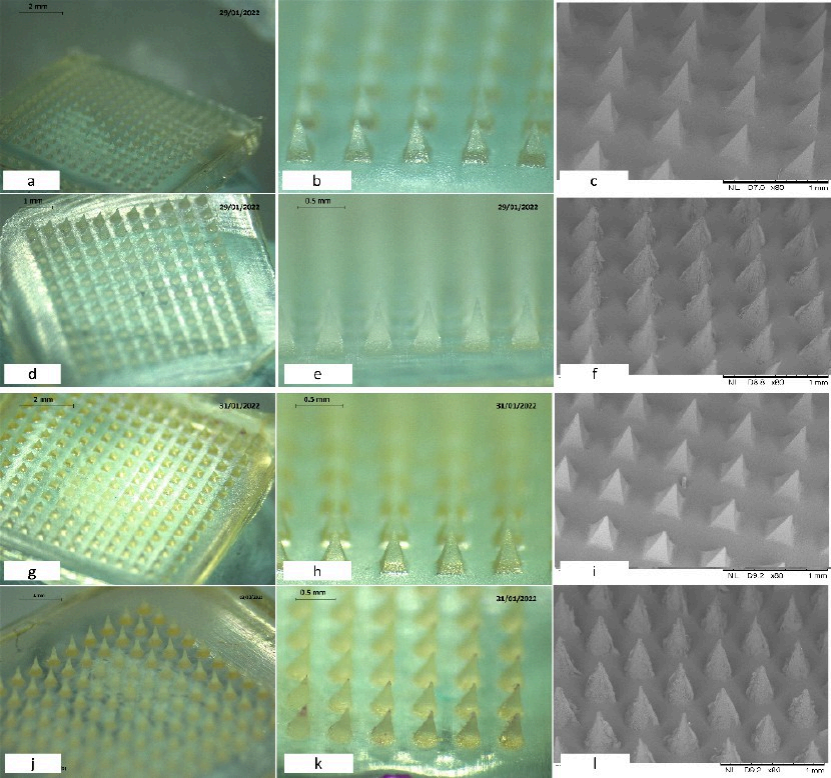
Morphology of the MNs made of F1 (a–f) and F2 (g–l) using 11 × 11 and 14 × 14 molds obtained by optical microscopy and SEM. F1, cross-linked with citric acid; F2, cross-linked with PEGdiacid.

Using the formulations and molds detailed in Table 9, MNs were produced. For clarity, formulations cross-linked with PEGdiacid were labeled F2, and those cross-linked with citric acid were labeled F1 as a control. The morphologies of the MNs formed from F1 and F2 via 11 × 11 and 14 × 14 molds are depicted in Figure 14, with a distinct geometry post cross-linking. The 11 × 11 MNs, characterized as conical, have a height of approximately 600 μm, interspaced precisely at 300 μm, and a base width of 300 μm, ensuring uniform geometry. In contrast, the 14 × 14 microneedles display a pyramidal structure post-cross-linking, reaching a height of approximately 600 μm, with the interspacing reduced to 250 μm for a more closely packed configuration. The pyramidal microneedles have a base width of 450 μm, providing enhanced stability. The MNs derived from formulations F1 and F2 had well-defined shapes, consistent heights, and precisely controlled interspacing.

**Table 9:** Composition and Processing Parameters of Polymeric Aqueous Blends for MNs^a^

formulation no.	type of hydrogel	composition	thermal treatment	MN mold geometry
F1	PVA/PVP/citric acid	Citric acid (1.5% w/w), PVP (10% w/w) and PVA (15% w/w)	130 °C for 40 min	11 × 11, conical, height 600 μm, interspacing 300 μm, base width 300 μm
F2	PVA/PVP/PEGdiacid	PEGdiacid (2.25% w/w), PVP (10% w/w) and PVA (15% w/w)	150 °C for 20 min	14 × 14, pyramidal, height 600 μm, interspacing 250 μm, base width 450 μm

a F1, crosslinked with citric acid; F2, crosslinked with PEGdiacid.

### 3.6 Mechanical Strength Indication of MNs

**Figure 15:**
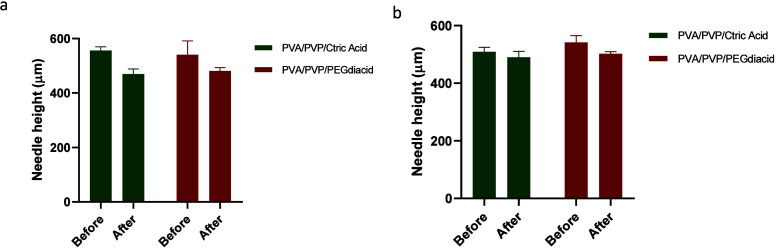
Results of the height reduction from compression of MNs using 11 × 11 molds (a) and 14 × 14 molds (b) (Means + SDs, *n* = 5). PVA/PVP/Citric acid: cast from aqueous blend containing citric acid (1.5% w/w), PVP (10% w/w) and PVA (15% w/w) cross-linked at 130 °C for 40 min. PVA/PVP/PEGdiacid: cast from aqueous blend containing PEGdiacid (2.25% w/w), PVP (10% w/w) and PVA (15% w/w) cross-linked at 150 °C for 20 min.

Each MN, derived from formulations F1 and F2 using 11 × 11 and 14 × 14 molds, was subjected to a compression force of 32 N against a solid surface. The needle heights were meticulously assessed through optical microscopy before and after compression, and the resulting height reduction data are presented in Figure 15. Comparing the height reduction percentages of MNs produced with 11 × 11 molds, F1 and F2 demonstrated reductions of 15.52 and 11.45%, respectively. In contrast, MNs created from 14 × 14 molds exhibited lower height reduction percentages, with values of 3.74 and 7.67% for F1 and F2, respectively (*n* = 5). The 14 × 14 mold, housing a total of 196 needles, differed significantly from the 11 × 11 mold with 121 needles, impacting the force distribution per needle. The higher needle count in the 14 × 14 mold resulted in less force being exerted on each needle, leading to a diminished percentage of height reduction. Pyramid-shaped needles provided a more uniform stress distribution, enhancing structural integrity by spreading the load across multiple sides and preventing concentration at a single point. This characteristic improved the overall durability of the MNs. The height reduction percentages for all formulations remained within the 20% range, indicating that the microneedles possessed the desired hardness and mechanical strength to penetrate the skin without fracturing.^34,54^

### 3.7 Insertion Ability of MNs

**Figure 16:**
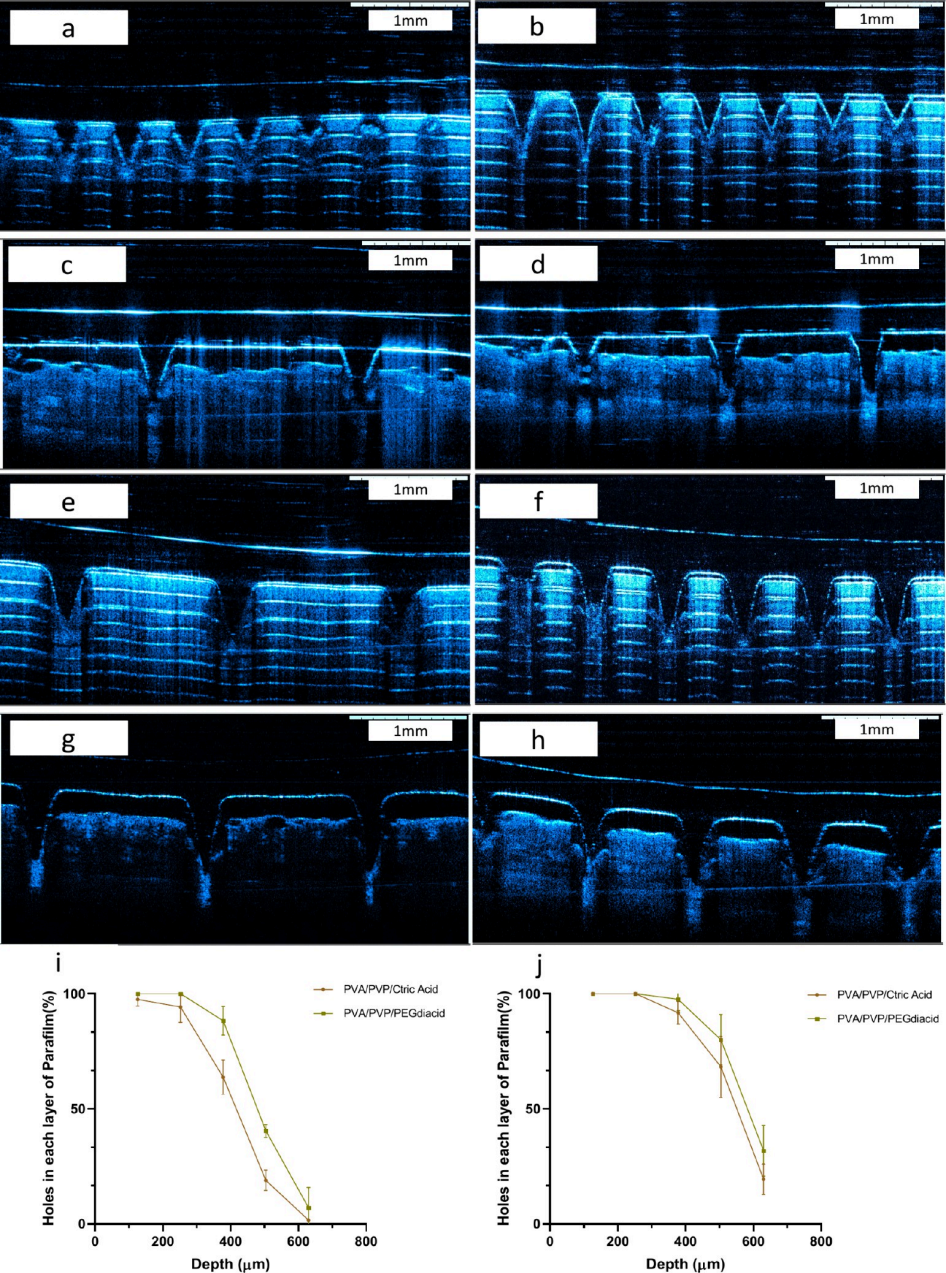
Representative OCT images depicting the insertion of MNs using 11 × 11 (a–d) and 14 × 14 mold (e–h) in Parafilm (a, b, e, f) and skin (c, d, g, h) for F1 (a, c, e, g) and F2 (b, d, f, h). Results of the MNs Parafilm insertion test from 11 × 11 molds (i) and 14 × 14 molds (j) (Means ± SDs, *n* = 5). PVA/PVP/citric acid: cast from aqueous blend containing citric acid (1.5% w/w), PVP (10% w/w), and PVA (15% w/w) cross-linked at 130 °C for 40 min. PVA/PVP/PEGdiacid: cast from aqueous blend containing PEGdiacid (2.25% w/w), PVP (10% w/w), and PVA (15% w/w) cross-linked at 150 °C for 20 min.

The determination of the insertion depth following the application of MNs was conducted via optical coherence tomography (OCT). MNs were inserted into a validated artificial skin model comprising Parafilm and neonatal pig full-thickness skin (750 μm). Eight layers of Parafilm were folded to achieve a total height of approximately 1008 μm, with each sheet having a thickness of approximately 126 μm. Upon insertion, the microscopic examination of each layer revealed the holes created by the MAPs, and the hole percentage was calculated. As illustrated in Figure 16i,j, MNs produced from a 14 × 14 mold were inserted to a maximum depth between 504 and 530 μm for F1 and F2 (Figure 16e,f). The insertion depths for the MNs of F1 and F2 from an 11 × 11 mold ranged from 378 to 504 μm (Figure 16a,b). ImageJ analysis indicated that MNs manufactured from 14 × 14 molds exhibited penetration depths of 508 and 522 μm in full-thickness neonatal porcine skin for F1 and F2, respectively (Figure 16g,h). For the 11 × 11 molds, the insertion depths of the MAPs from F1 and F2 were measured at approximately 454 and 491 μm (Figure 16c,d). The acquired insertion depths from the skin and Parafilm insertions mutually confirmed each other. Importantly, all formulations surpassed the thickness of the SC (10–50 μm), indicating that the MAPs possessed the ability to penetrate the SC of the skin.^55,56^

### 3.8 Development of Drug Reservoirs

**Figure 17:**
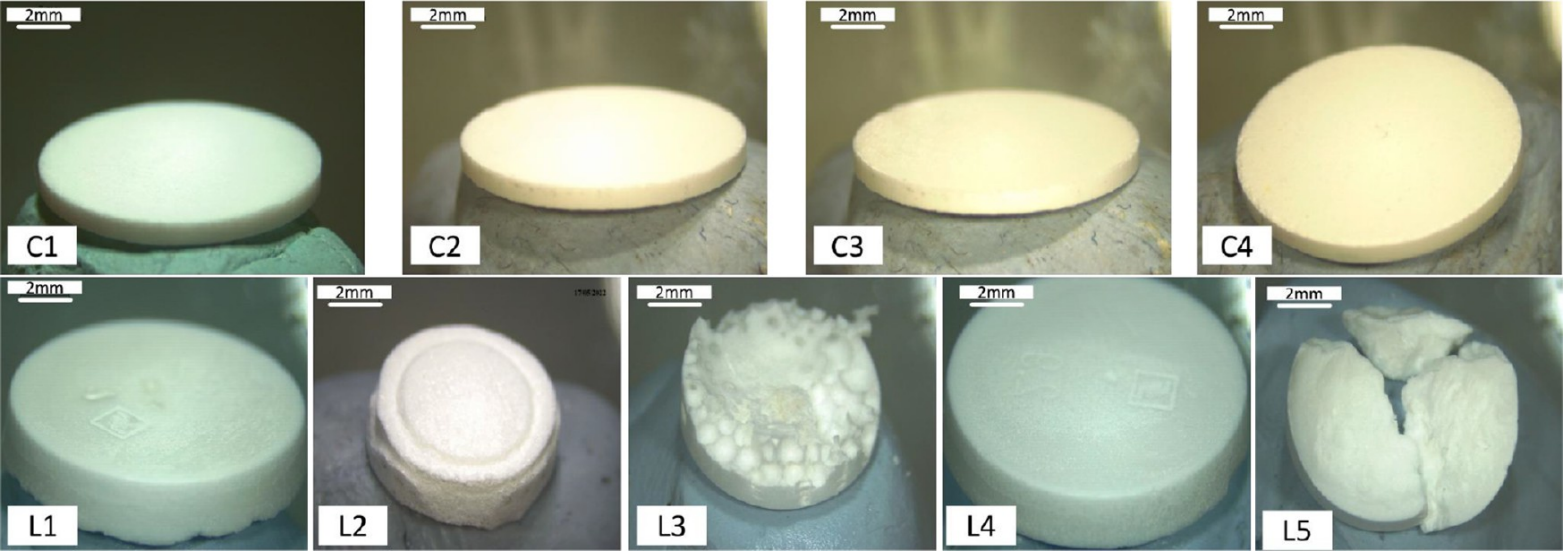
Physical appearance of the direct compressed tablet (C1–C4) and lyophilized wafer (L1–L5) of enfuvirtide. C1 and C3:5% enfuvirtide, 85% mannitol, 10% gelatin; compressed at 2 and 4 tons. C2 and C4:5% enfuvirtide, 90% mannitol, 5% gelatin; compressed at 2 and 4 tons. L1-L5:5% enfuvirtide, with decreasing mannitol (20% in L1, 10% in L2, 2.5% in L3-L5) and gelatin (5, 2.5, and 1.25% in L3–L5). All formulations were lyophilized under the same conditions.

Enfuvirtide reservoir development involved the use of lyophilization and compression techniques. Figure 17 shows that all directly compressed tablets (C1–C4) exhibited consistent shapes, sizes, and colors with smooth surfaces, regardless of the formulation or compression force. In contrast, freeze-dried reservoirs showed variation. L1, L2, and L4 had a uniform appearance with intact surfaces and good structural integrity, whereas L3 displayed a honeycomb-like surface. L5 exhibited poor structural integrity and crumbled during demolding, possibly due to the lack of a binding agent. Consequently, further investigations were conducted on all the tablets and L1–L4 wafers.

The performance of the reservoirs, including the dissolution time, hardness, and drug recovery, is systematically recorded in Table 10. The incorporation of a greater percentage of mannitol in the formulation, coupled with the compact structure of compressed tablets, resulted in an extended dissolution duration ranging from 5 to 7 min, with compression force exerting a significant influence. In contrast, the direct sublimation of water from ice to vapor during the freeze-drying process yielded a porous microstructure in the wafer, expediting the rehydration process.^57^ Mannitol, which serves as both a bulking agent and cryoprotectant in the formulations, not only contributed to the aesthetic appearance and mechanical robustness of the freeze-dried products but also mitigated ice formation around the enfuvirtide. Additionally, mannitol plays a crucial role in maintaining the structural integrity of the drug by increasing formulation viscosity and limiting the movement of enfuvirtide.^58^ However, a greater proportion of mannitol substantially impeded dissolution, as evidenced by the significantly prolonged dissolution time of L1 and L2 than that of L3 (*p* < 0.001). This hindrance was attributed to the formation of crystalline barriers and alterations in porosity, collectively resulting in delayed dissolution. Furthermore, an increased concentration of mannitol led to decreased wafer hardness and greater variation in drug amount, potentially influenced by the inconsistent distribution of gelatin at elevated mannitol concentrations. The comprehensive analysis indicated that the lyophilized L4 wafer rapidly dissolved, had an acceptable hardness, and exhibited favorable drug recovery. Consequently, it was selected as the final drug reservoir for integration with MNs.

**Table 10:** Dissolution Time, Hardness, and Drug Recovery of the Reservoirs (Means ± SDs, *n* = 5)

	composition (% w/w)							
formula ID.	enfuvirtide	mannitol	gelatin	water	processing	dissolution time (s)	hardness (N)	drug recovery (%)
C1	5	85	10	NA	2 N compression	275 ± 12	32	92.51 ± 7.25
C2	5	90	5	NA	2 N compression	266 ± 37	32	95.78 ± 3.81
C3	5	85	10	NA	4 N compression	340 ± 11	32	92.66 ± 10.22
C4	5	90	5	NA	4 N compression	322 ± 19	32	97.24 ± 3.73
L1	5	20	5	70	freeze-drying	45 ± 27	<32	79.40 ± 15.59
L2	5	10	5	80	freeze-drying	28 ± 16	32	90.28 ± 19.18
L3	5	2.5	5	87.5	freeze-drying	13 ± 6	32	95.28 ± 12.99
L4	5	2.5	2.5	90	freeze-drying	3 ± 1	32	99.33 ± 5.25
L5	5	2.5	1.25	91.25	freeze-drying			

#### 3.8.1 Characterization of the Chosen Reservoir

The lyophilized wafers, composed of 5% enfuvirtide, 2.5% mannitol, 2.5% gelatin, and 90% w/w water in the aqueous formulation, exhibited a hardness of 42 N and could be demolded completely. The cylindrical wafers, which are 12.2 mm in diameter and 2 mm in height, feature a flat, smooth surface without cracks or defects. They demonstrated consistent weight (24.5 mg) and content uniformity (11.05 mg) with a variation of less than 5% in both the intra- and interbatch analyses. The organoleptic evaluation revealed no unpleasant odor. The moisture content of the wafers was maintained below 0.1%, ensuring stability and inhibiting microbial growth. After one month of storage in an airtight glass container covered with aluminum foil at ambient temperature, enfuvirtide content did not significantly change (*p* > 0.05). In conclusion, the lyophilized wafer exhibited uniform physical characteristics, favorable organoleptic properties, low moisture content, and stability, collectively contributing to its effectiveness when used in combination with MNs.

#### 3.8.2 Antiviral Activity and Cytotoxicity of Enfuvirtide Wafer

The cell concentration and viability were evaluated with representative images of MT4 cell distribution, diameter, total number of cells dyed with OA, and number of dead cells dyed with DAPI displayed in Figure S7. The viability was calculated with eq 12, where %viability was the percentage of viable cells in the cell suspension, *C_t_* was the total concentration of cells, and *C*_nv_ was the concentration of nonviable cells. The viability of MT4 was 97.8% with a concentration of 1.32 × 10^6^ cells/mL. A seeding concentration of 6 × 10^5^ cells/mL was prepared from this stock by pelleting and resuspending the cells. The cell viability and concentration were deemed appropriate for HIV infection studies, ensuring optimal conditions for efficient viral binding, entry, and replication while maintaining a balanced healthy and proliferative state.
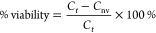
12

The infectivity of the HIV-1 NL4–3 strain was assessed by the titration of MT4 cells, and syncytium formation was observed via microscopy to evaluate the effect of HIV-1 infection. Results are shown in Figure S8, which confirms the quality and suitability of the virus stock for use in this study. Furthermore, consistent and reproducible CPEs were observed in the infected cells across both titration plates, validating the virus strain’s infectivity, which is crucial for obtaining accurate and reliable data in studies on HIV-1 replication and pathogenesis.^59^ A viral concentration sufficient to completely lyse the cells was used in this study. Consequently, a 4-fold concentration of the 1250-fold diluted virus (approximately 300-fold dilution) was selected as the infective concentration, considering the dilution factor introduced by the addition of the compound culture medium in the antiviral experiment. The antiviral activities of the formulations loaded with enfuvirtide or enfuvirtide were determined at 5 days in MT4 cells against HIV-1 NL4.3. The potential cytotoxicity of enfuvirtide and the compounds used in MN fabrication was investigated together with antiviral efficacy studies. The formazan produced by viable cells and the syncytium was observed via optical microscopy (Figure S9). The antiviral activities of the formulations loaded with enfuvirtide or enfuvirtide were determined at 5 days in MT4 cells against HIV-1 NL4.3. The potential cytotoxicity of enfuvirtide and the compounds used in MN fabrication was investigated together with antiviral efficacy studies. The formazan produced by viable cells and the syncytium is shown in Figure S9.

**Figure 18:**
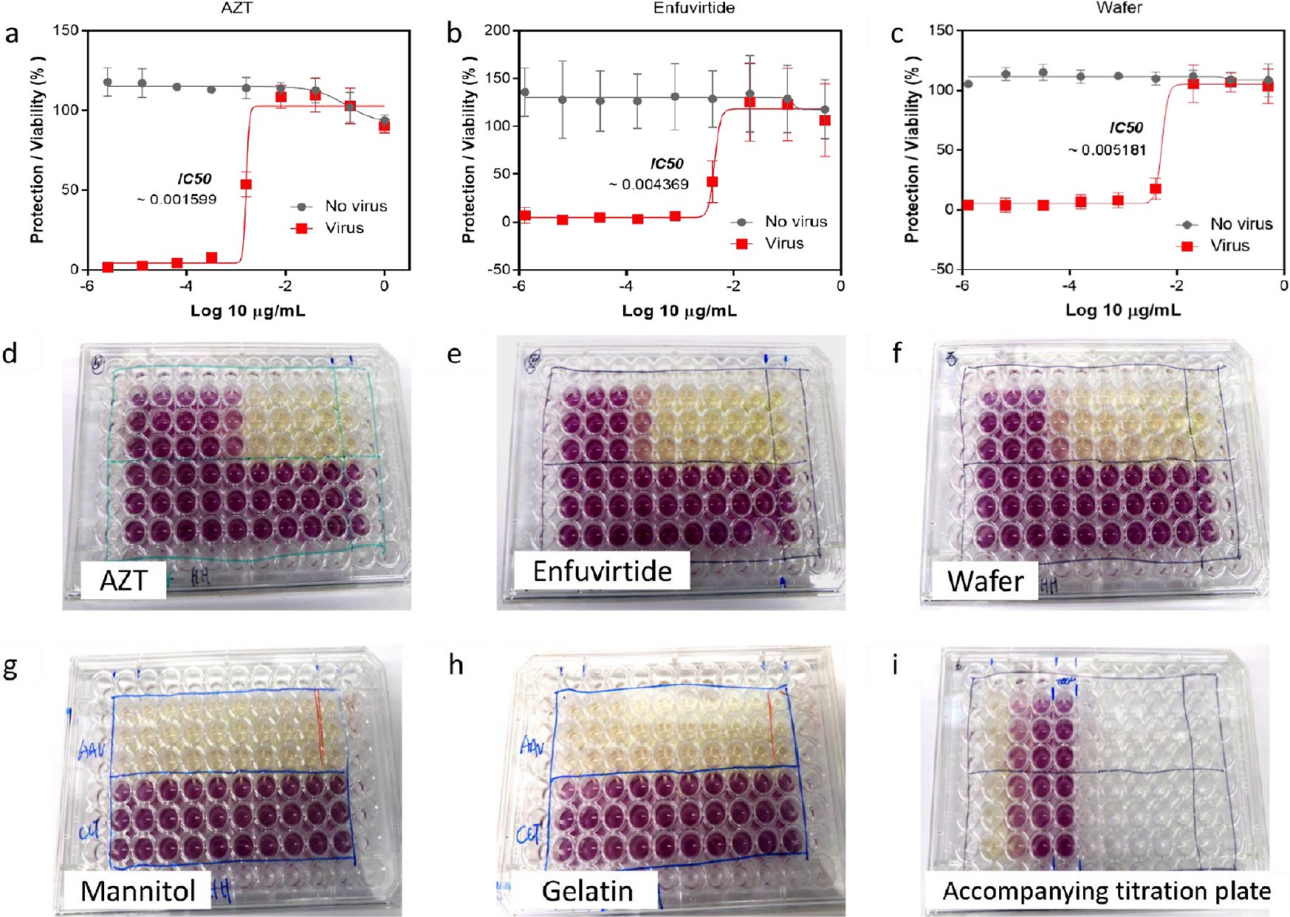
Dose–response curves AZT (a), enfuvirtide (b), and enfuvirtide loaded into wafers (c)(Means ± SDs, *n* = 3), antiviral activity and cytotoxicity plates AZT (d), enfuvirtide (e), wafer (f), mannitol (g), gelatin (h), and accompanying titration plates (i). Purple stands for viable cells and yellow stands for dead cells.

The 96-well plates were separated into upper and lower sets for antiviral activity and cytotoxicity, respectively. AZT was utilized as a positive control for HIV inhibition in MT4 cells, yielding an IC_50_ of 1.6 ng/mL, which aligns closely with previously reported values (1.87 ng/mL), thus validating the results of the correctness experiment (Figure 18a,d).^60^ After the appropriate IC_50_ range from the initial antiviral assay was determined, the starting concentration for enfuvirtide was adjusted from 15 to 0.5 μg/mL to ensure the IC_50_ would appear in the middle of the plate, promoting uniform dosage exposure and consistency across different plates. As shown in Figure 18b,c,e,f, enfuvirtide, as well as enfuvirtide loaded into lyophilized wafers, inhibited viral replication with similar IC_50_ values (*p* > 0.05), indicating that the potency of enfuvirtide was preserved throughout the manufacturing process. As shown in Figure 18g,h, the excipients mannitol and gelatin exhibited no cytotoxicity or antiviral activity in this study. This was evidenced by similar cell viability in the lower set (wells 2E to 2G) compared with the negative control (wells 11E to 11G), indicating that the materials were nontoxic to MT4 cells at the highest concentrations tested (Table 11). This finding was crucial for ensuring accurate and reliable results in subsequent experiments, as cell death or toxicity could confound data interpretation. Furthermore, no significant changes in cell morphology or behavior were observed at the highest concentrations, further supporting the safety of these polymers and excipients.

**Table 11:** IC_50_ and CC_50_ of AZT, Enfuvirtide, and Enfuvirtide Loaded into Wafers and Excipients against NL4-3 HIV-1-Infected MT4 Cells (*n* = 3)

compound	IC_50_ (ng/mL)	CC_50_ (μg/mL)
AZT	1.599	>1
enfuvirtide	4.369	>15
wafer	5.181	>15
mannitol	>15	>15
gelatin	>15	>15

### 3.9 Ex Vivo Enfuvirtide Permeation from MN Associated with the Reservoir

**Figure 19:**
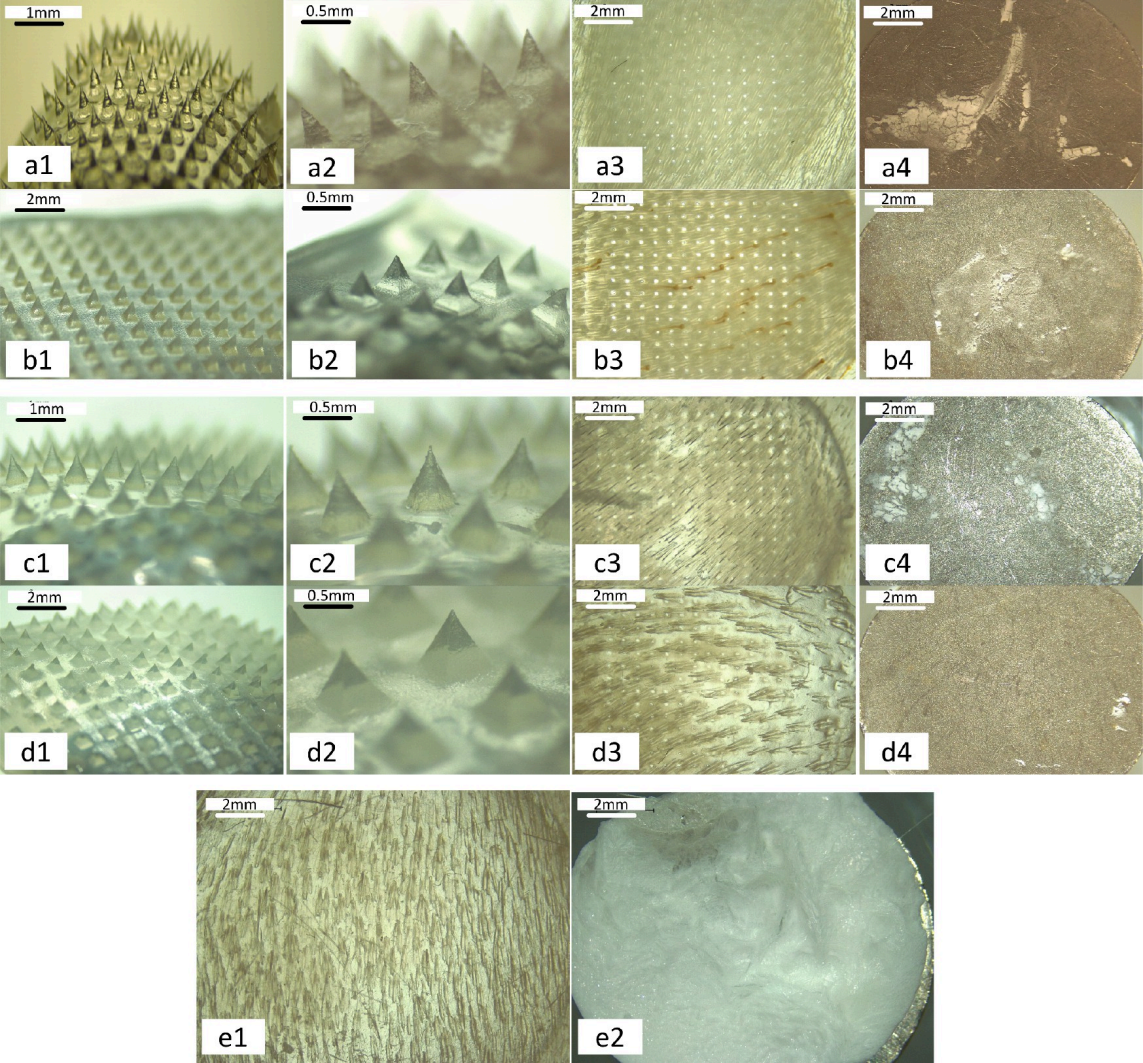
MN, skin, and wafer components of 11 × 11 (a1–4, c1–4) and 14 × 14 (b1–4, d1–4) MNs from formulations F1 (a, b), F2 (c, d), and the control (e1–2) after *ex vivo* permeation. F1, cross-linked with citric acid; F2, cross-linked with PEGdiacid.

Enfuvirtide permeation from MNs with reservoirs was investigated ex vivo via Franz cells. After 24 h, optical microscopy revealed the morphology of the MNs, skin, and reservoirs, as depicted in Figure 19. Needles produced from F1 and F2 using 11 × 11 and 14 × 14 molds remained intact with defined shapes and consistent heights post swelling, indicating sufficient mechanical strength for removal post administration (Figure 19a1-2, b1–2, c1–2, d1–2). Discernible holes were created by the insertion of MNs, and the numbers were in accordance with the mold matrix (Figure 19a3,b3,c3,d3). The residual reservoirs had minimal effects on the metal weight postdissolution (Figure 19a4,b4,c4,d4), in contrast with the undissolved reservoirs in the control group (Figure 19e1-2), highlighting the role of MNs in compromising skin barriers.^61^

**Figure 20:**
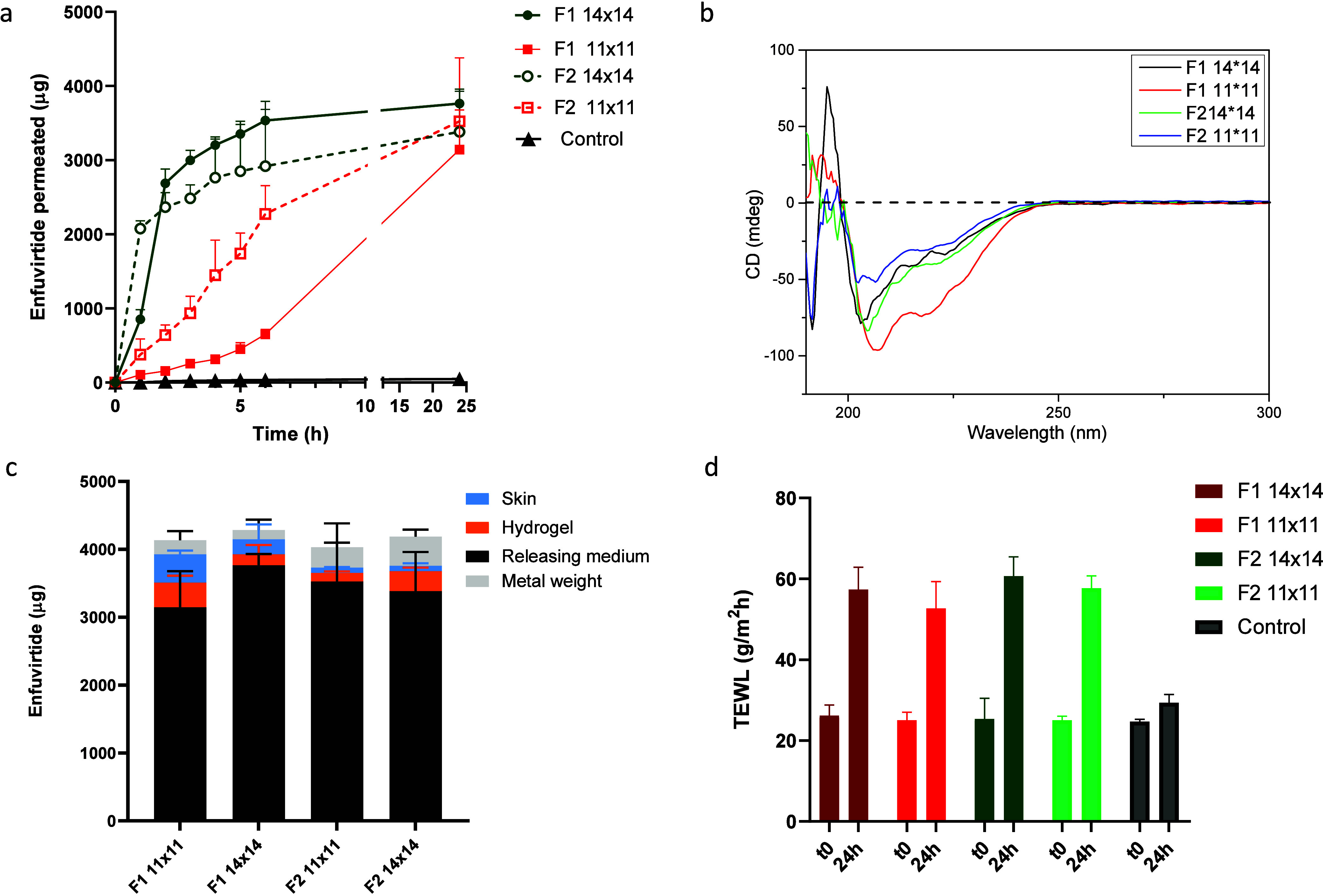
Permeation profiles (a), circular dichroism spectrum (b), and distribution (c) of enfuvirtide and TEWL values (d) of the skin after 24 h of ex vivo study (means + SDs, *n* = 4). F1, cross-linked with citric acid; F2, cross-linked with PEGdiacid.

The cumulative amount of enfuvirtide permeated in 24 h is presented in Figure 20a. Notably, a substantial increase in the permeation of enfuvirtide within the initial 2 h was observed in 14 × 14 molded MNs. From 6 h onward, a significant disparity existed in the cumulative permeation of enfuvirtide between 11 × 11 and 14 × 14 molded MNs for both formulations (*p* < 0.05). Beyond this time point, enfuvirtide permeation increased gradually, and after 24 h, the cumulative permeation among all four MNs was statistically similar (*p* > 0.05). The amounts were 3764.49 ± 165.40 (34.07% ± 1.50%), 3144.14 ± 533.48 (28.45% ± 4.83%), 3382.24 ± 578.23 (30.61% ± 5.23%), and 4006.345 ± 266.85 (36.26% ± 2.41%) for F1 14 × 14, F1 11 × 11, F2 14 × 14, and F2 11 × 11, respectively. The permeation percentage of enfuvirtide using the PVA-based hydrogel system with a lyophilized wafer in this study was significantly higher than that of atorvastatin and pyrazinamide, which were reported to achieve less than 15 and 20% permeation, respectively.^29,62^ This highlights the feasibility and superiority of this strategy. After 24 h, the swelling percentage was 461.21% ± 16.32% (*n* = 6) for F1 and 487.22% ± 27.39% for F2, with no significant difference noted. During the early stage of permeation, enfuvirtide release was primarily governed by swelling. Once fluid traverses the microconduits in the hydrogel matrix and reaches the reservoir, it dissolves and releases the drug. When swelling nearly reached equilibrium in 24 h, permeation was no longer confined to the hydrogel kinetics, thereby explaining the similarities in cumulative permeation at that point. The permeation profiles of F1 and F2 from x molded MNs were similar, characterized by a rapid increase in the first 2 h followed by a steady increase until reaching a plateau, indicative of first-order release kinetics. Conversely, the permeation of enfuvirtide from F1 and F2 from 11 × 11 MNs followed zero-order release kinetics, which was evident at a constant rate.

The α-helix of enfuvirtide is essential for its insertion into the viral membrane to prevent fusion with cell membranes. For the conformation of the secondary structure of this peptide drug, a CD spectrum was obtained from the analysis of the diluted sample from the reservoir compartment (Figure 20b). The two minima at approximately 208 nm (π–π*) and 222 nm (n−π*) and the maximum at 191–193 nm (π–π*) confirmed the presence of an α-helix within the structure of the enfuvirtide in the ex vivo study, indicating that the drug maintained its efficacy after manufacture and permeation.^15^ Upon completion of the ex vivo study, the enfuvirtide content within the MNs, porcine skin samples, and reservoirs was quantified (Figure 20c). It was evident that a majority of the enfuvirtide permeated into the release medium, and no significant difference was observed in the distribution of enfuvirtide in the hydrogel, skin, or metal weight between the F1 and F2, showcasing the comparability of the hydrogel formulations between the novel formulation and the control system (*p* > 0.05). TEWL analysis was carried out on vertical Franz diffusion cells before and after *ex vivo* studies. The TEWL value of untreated skin was recorded as t0 and was compared to the TEWL value of the skin treated with MNs after 24 h, where significant differences were observed in all the dermatomed skin samples (*p* < 0.0001) from approximately 23 g/m^2^ h to above 55 g/m^2^ h, indicating that the MNs disrupted the integrity of the epidermis through permeation (Figure 20d). In contrast, the TEWL value of the skin of the control group did not significantly differ, which implied that the structural completeness of the skin was in contact with the release medium over 24 h (*p* = 0.56).

## 4 Conclusions

This study provides a comprehensive investigation into the design and optimization of hydrogel-forming MNs using a QbD framework. By systematically identifying and controlling CMAs and CPPs, as well as understanding their interactions, we successfully enhanced the mechanical properties and drug delivery performance of MNs, establishing a foundation for further optimization and clinical translation. For the first time, PEGdiacid, a biocompatible cross-linker, was utilized to form hydrogels with PVA and PVP, demonstrating its suitability for therapeutic applications. The integration of a lyophilized enfuvirtide reservoir with gelatin and mannitol facilitated efficient dissolution while preserving the drug’s efficacy. Ex vivo permeation studies further highlighted the adaptability of MNs in modulating drug delivery through variations in the needle geometry.

These findings underline the potential of hydrogel-forming MNs as a minimally invasive, patient-friendly transdermal delivery strategy for enfuvirtide. Additionally, the insights gained from this work could contribute to the development of regulatory guidelines and their inclusion in international pharmacopoeias. Future research will focus on in vivo validation, scalable manufacturing, and real-time feedback systems to ensure consistent dosing across diverse skin types, facilitating regulatory approval and broader clinical adoption.

## Data Availability

The raw data required to reproduce the above findings are available on request.

## Supporting Information Available

Figure S1: Schematic of HMAP application in combination with reservoirs; Figure S2: Illustration of side-by-side horizontal diffusion cells set up for in vitro permeation studies and enfuvirtide diffusion studies; Figure S3: Rheological characterization of hydrogels via oscillatory shear with parallel plates; Figure S4: Schematic illustration of HMAP fabrication; Figure S5: Schematic diagram of the preparation of enfuvirtide tablets; Figure S6: Schematic illustration of the modified Franz cell system for ex vivo permeation studies; Figure S7: Representative image of cells dyed with OA and DAPI (a), cell diameter distribution (b), total number of cells (c) and number of dead MT4 cells (d); Figure S8: Titration plate (a1), an abstract titration plate with an OD reading (a2) and a representative formation of syncytium from column 5 (b); Figure S9: Representative images of nonviable (a), partially viable (b) and viable (c) MT4 cells infected with HIV in the wells and the formation of syncytia in nonviable cells (d); Table S1: Overview of therapeutic peptides being studied for transdermal delivery using microneedles; Table S2: Examples of DoE applications in pharmaceutical product development; Table S3: Experimental design observations for PVA/PVP/PEGdiacid–based hydrogel formulations; Table S4: Freeze-drying procedure for the lyophilized wafers; and cell viability evaluation procedure virus titration process (PDF)
