# Correction to “Quality by Design-Guided Development of Hydrogel-Forming Microneedles for Transdermal Delivery of Enfuvirtide”

**DOI:** 10.1021/acsami.5c20308

**Published:** 2025-10-20

**Authors:** Huanhuan Li, Lalitkumar K. Vora, Qonita Anjani, Abraham M. Abraham, Yilin Cong, Natalia Moreno-Castellanos, Ester Ballana, Eva Riveira Muñoz, Maria Nevot, Ryan F. Donnelly

In the original paper, the authors regret that an error occurred during the assembly of C2 and C3 in Figure 17. Specifically, C2 and C3 are the same image of C2. The corrected version of Figure 17 is provided below. This correction does not affect the description, interpretation, or conclusions of the study.

**Figure 17:**
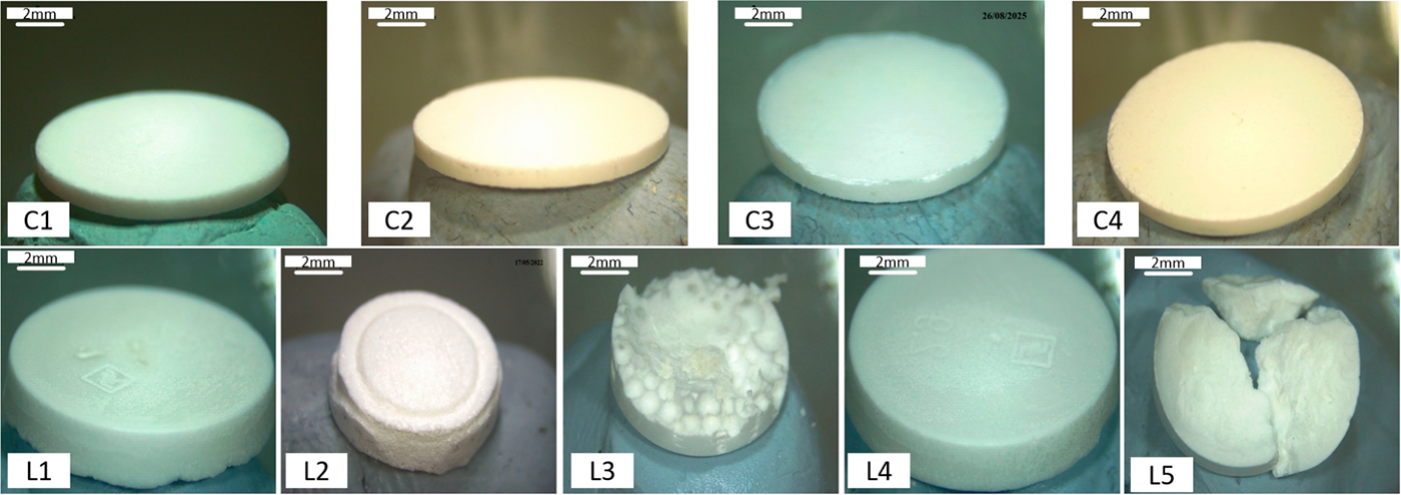
Physical appearance of the direct compressed tablet (C1–C4) and lyophilized wafer (L1–L5) of enfuvirtide. C1 and C3:5% enfuvirtide, 85% mannitol, 10% gelatin; compressed at 2 and 4 tons. C2 and C4:5% enfuvirtide, 90% mannitol, 5% gelatin; compressed at 2 and 4 tons. L1-L5:5% enfuvirtide, with decreasing mannitol (20% in L1, 10% in L2, 2.5% in L3-L5) and gelatin (5, 2.5, and 1.25% in L3–L5). All formulations were lyophilized under the same conditions.

