# Supplementary material for: Quality by Design-Guided Development of Hydrogel-Forming Microneedles for Transdermal Delivery of Enfuvirtide
Source: ACS Appl Mater Interfaces. 2025 Mar 10;17(18):26227–51. doi: 10.1021/acsami.5c00499 (PMC12067383; doi:10.1021/acsami.5c00499)
Supplement: Supplementary file 1 — am5c00499_si_001.pdf [file am5c00499_si_001.pdf]

Supporting Information

Quality by design guided development of hydrogel-forming microneedles for transdermal delivery of enfuvirtide

Huanhuan Li[1], Lalitkumar K. Vora[1], Qonita Anjani[1], Abraham M. Abraham[1], Yilin Cong[1], Natalia Moreno-Castellanos[2], Ester Ballana[3], Eva Riveira Muñoz[3], Maria Nevot[3], Ryan F. Donnelly [1*]

1. School of Pharmacy, Queen's University Belfast, BT9 7BL, United Kingdom
2. Basic Science Department, Faculty of Health, Universidad Industrial de Santander, Bucaramanga 680001, Colombia
3. AIDS Research Institute—IrsiCaixa, 08916, Spain

* Corresponding author
E-mail address: r.donnelly@qub.ac.uk (Ryan Donnelly)

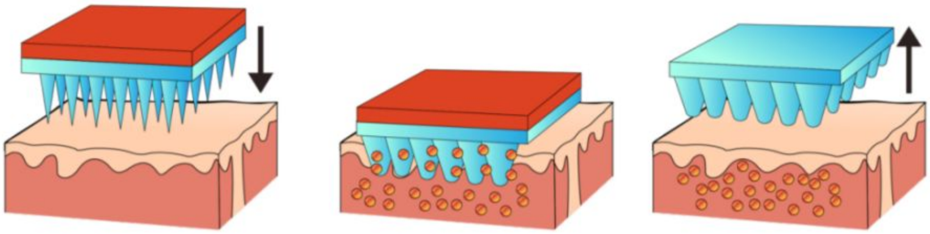

Figure S1. Schematic of HMAP application in combination with reservoirs.

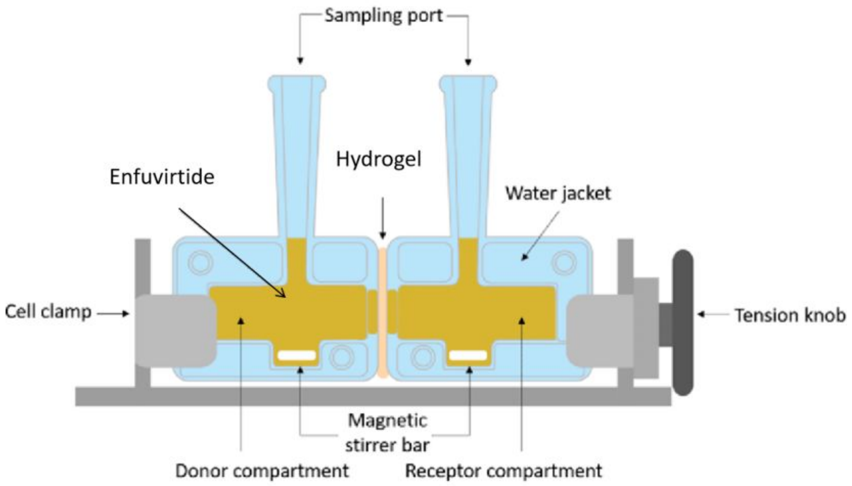

Figure S2. Illustration of side-by-side horizontal diffusion cells set up for *in vitro* permeation studies and enfuvirtide diffusion studies.

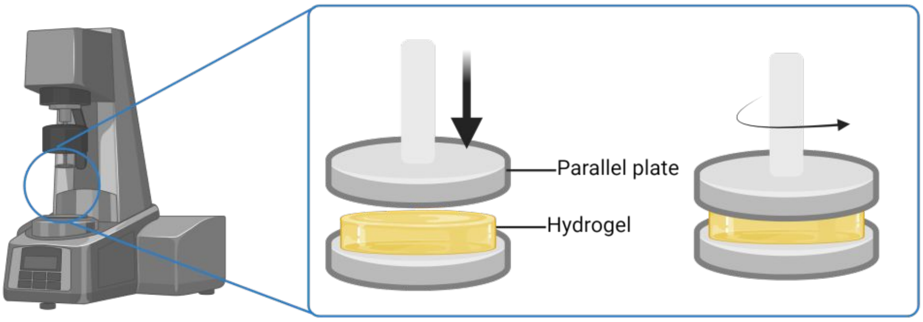

Figure S3. Rheological characterisation of hydrogels *via* oscillatory shear with parallel plates.

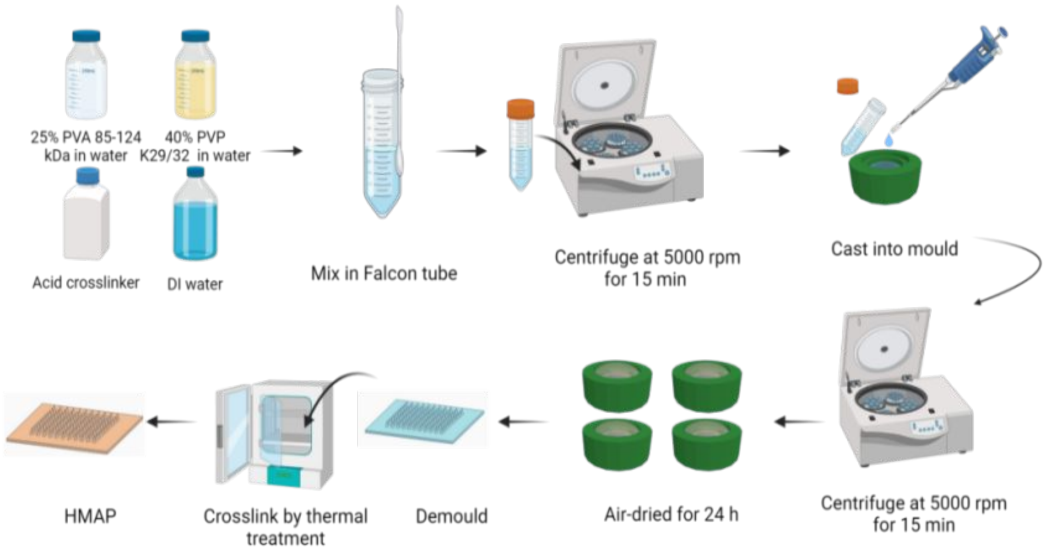

Figure S4. Schematic illustration of HMAP fabrication.

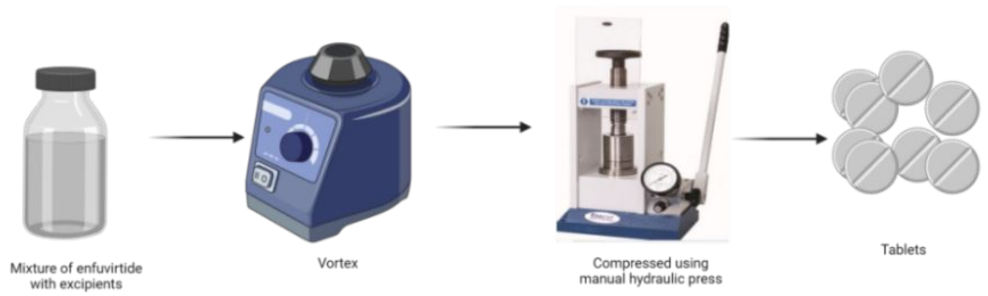

Figure S5. Schematic diagram of the preparation of enfuvirtide tablets.

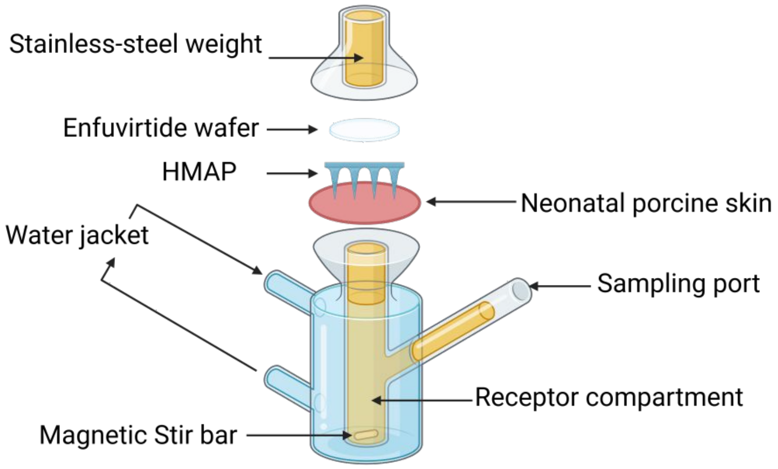

Figure S6. Schematic illustration of the modified Franz cell system for *ex vivo* permeation studies.

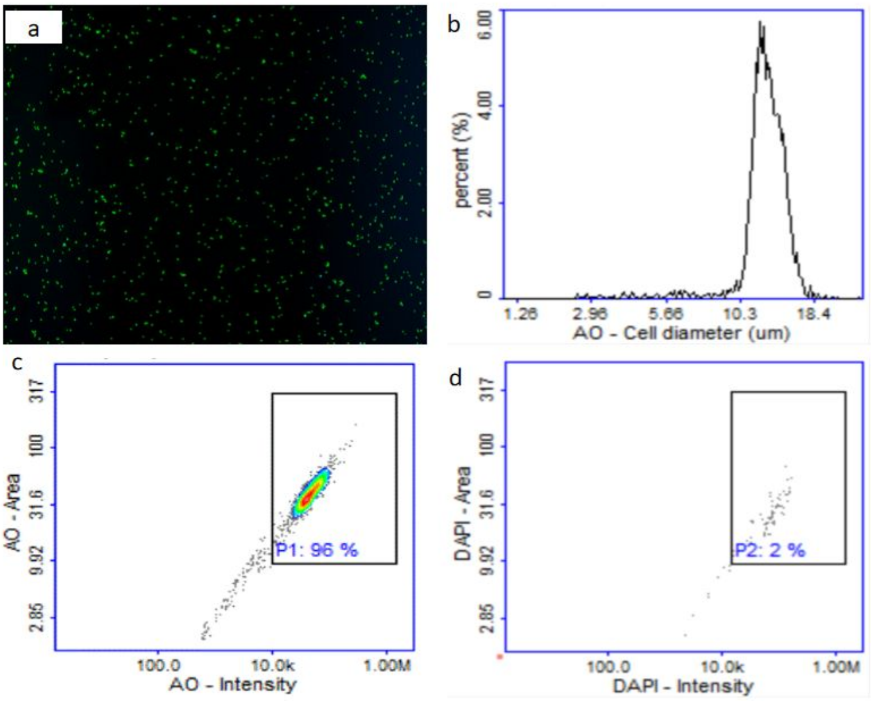

Figure S7. Representative image of cells dyed with OA and DAPI (a), cell diameter distribution (b), total number of cells (c) and number of dead MT4 cells (d).

a1

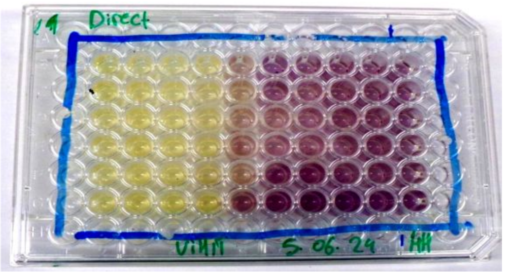

a2

Times of the virus dilution		10	50	250	1250	6250	31250	156250	781250	3906250	no virus	
Absorbance at 550 nm												
row/column	1	2	3	4	5	6	7	8	9	10	11	12
A												
B		0.121	0.115	0.18	0.165	0.403	0.472	0.536	0.531	0.488	0.53	
C		0.126	0.158	0.207	0.204	0.328	0.527	0.535	0.555	0.544	0.525	
D		0.125	0.162	0.203	0.203	0.419	0.467	0.525	0.633	0.606	0.531	
E		0.144	0.138	0.199	0.185	0.417	0.482	0.537	0.57	0.554	0.545	
F		0.137	0.156	0.19	0.252	0.353	0.534	0.535	0.537	0.575	0.565	
G		0.138	0.134	0.122	0.132	0.454	0.508	0.556	0.681	0.575	0.622	
H												

b

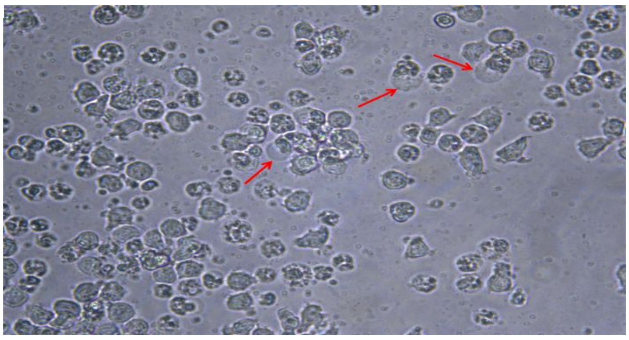

Figure S8. Titration plate (a1), an abstract titration plate with an OD reading (a2) and a representative formation of syncytium from column 5 (b).

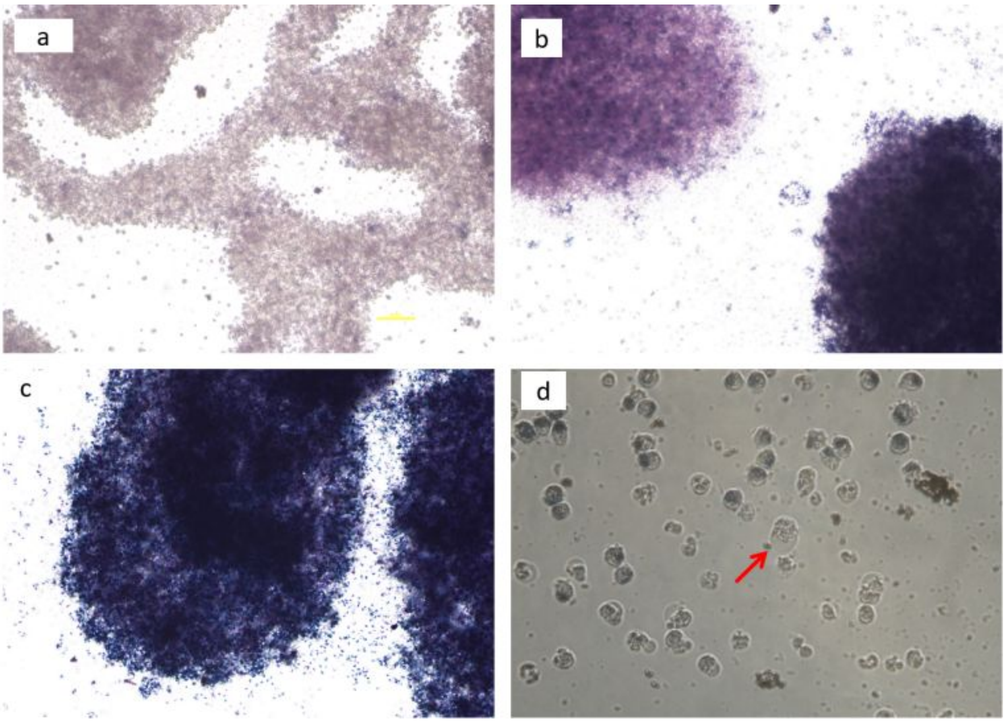

Figure S9. Representative images of nonviable (a), partially viable (b) and viable (c) MT4 cells infected with HIV in the wells and the formation of syncytia in nonviable cells (d).

Table S1. Overview of therapeutic peptides being studied for transdermal delivery using microneedles.

Peptide	Mw	Sequence	Dose	MN type	Size	Needle numbers	Skin model	Indication
Melanostatin	803.92 Da	PLG	–	Solid	700 µm length × 250 µm width	6	Human skin	Inhibited melanin formation
Rigin	959.04 Da	GQPR						Reduce inflammation
Palmitoyl-pentapeptide (Pal-KTTKS)	1191.06 Da	KXXKS	20 µL	Solid	150 µm-length and area 4 µm × 4 µm	121	Porcine ear skins	Anti-ageing
Tetrapeptide-3	456.6 Da	GQPR						Stimulate the dermal papilla
Hexapeptide	498.6 Da	VGVAPG						Anti-wrinkle and anti-ageing
Acetyl hexapeptide-3	889 Da	EEMQRR						Treat facial wrinkles
	1007.2 Da	CYXQNCPLG	0.37-2 µg	Coated	length of 470.2 ± 13.4 µm and base diameter 339.1 ± 16.3 µm	30, 15, 5	Human and mice skin	
Oxytocin M31		YVRPLWVRME						Vasodilator agent
WE14	–	WSRMDQLAKE LTAE						Antigen-specifc immunotherapy (ASI) of type 1 diabetes
Pro-insulin B9-23	–	SHLVEALYL-VCGERG						
Interferon-α-2b	–	–	23.79-4.94 µg	Dissolving	380 µm base length and 680 µm height	5, 6	Porcine skin	Treat chronic hepatitis B or C, hairy cell leukaemia
Ovalbumin	1773.9 Da	ISQAVHAAHAE INEAGR	96.6-11.0 µg	Solid	200–1000 µm long, 20–80 µm base diameter	192 × 8	Mouse skin	Antigen for immunization research
Botulinum toxin A (BT)	150–900 kDa	–	100 ng/ml	Hollow	670 × 340 × 65 µm	6	Human skin	Treatment of spastic and congenital entropion
β-galactosidase	800–1500 kDa	–	0.65 µg/ml					Enzymatic hydrolysis of lactose
polymyxin B	1203.5 Da	–	2.292mg	Dissolving	650 µm length, 200 µm base width	18 × 18	Porcine skin	Bactericidal activity against Gram-negative bacteria and a few Gram-positive species
Desmopressin	1069.22 Da	CYFQNCPRG	20 µg	Coated	200 µm long,	321 × 2	Guinea	Treatment of enuresis

Peptide	Mw	Sequence	Dose	MN type	Size	Needle numbers	Skin model	Indication
					170 µm width 35 µm thickness		pigs	
hGLuc mRNA and Lipo fectamine 2000 complex	>500 Da	-	0.2, 0.5 or 1.5 µg	Hollow	-	-	Porcine skin	Immunotherapeutic agents
BSA-FITC	66 kDa	-	-	Dissolving	600 µm height, 300 µm base width	12×12 and 19×19	Porcine skin	Model drugs for visualisation of the protein localisation
Proteolipid protein (PLP)	30 kDa	-	50 µg.ml^{-1}	Dissolving	600 µm length and diameter 125 µm or 200 µm	33×33	Pig skin	Trigger a specific immune response and improved neurological outcome in multiple sclerosis

Table S2. Examples of DoE applications in pharmaceutical product development.

Area	Application	Applied DoE Type
Oral drug delivery	Tablet formulation development	Multivariate design (fractional factorial design in 14 variables, 214-9 design, 35 experiments)
		Multivariate design + simplex optimisation by Modde Optimiser
		Fractional factorial designs (two studies); design space definition using a simplified Bayesian Monte Carlo simulation
		Mixture design
Oral drug delivery - immediate release (IR)	Dispersible tablets development	Several factorial experiments at 2-3 factors, 2-3 levels
	Immediate release tablet platform	Resolution V 25-1 fractional factorial design
	Fast dissolving pellets	25-1 fractional factorial design, 5 factors (4 numeric and 1 categorical), 2 levels
Oral drug delivery - modified release (MR)	Gastroretentive dosage form	3-level-3-factor, Box–Behnken design
Inhalation drug delivery	Powder for inhalation (formulation and process development)	Half-fractional factorial design with 5 factors at 2 levels with resolution V
		Face centered central Composite Design with 3 factors at 3 levels
		Risk assessment by Lean QbD Software
Transdermal drug delivery	Patch development	24 full factorial design
	Iontophoretic delivery	Face-centered central composite design
Cutaneous drug delivery (Topical)	Nanoemulsion for leishmaniasis (formulation development)	22 full factorial design
	Microsponge-based gel for surgical wounds (development)	3-factor, 3-level Box–Behnken design
Ocular drug delivery	PEGylated PLGA nanospheres (optimisation and characterisation)	Central composite factorial design
	Liquid crystalline nanoparticles (formulation optimisation)	Fractional factorial design 25-1; simplex-lattice experimental design
Injections	Formulation for parenteral nutrition (development)	D-optimal experimental design (mixture design)
Biopharmaceuticals	Antibody Formulation Robustness	Multivariate study (full factorial) including 2 factors at 2 levels
Nanopharmaceutics	Solid Lipid Nanoparticles for Inhalation (process development)	Two-level full factorial design (with no center points and three repetitions for each level)
Pharmaceutical processes	Dry powder inhaler capsule filling	D-optimal model with design statistics G-efficiency with three replicates
	Excipient micronisation	Full factorial (three variables at 2 levels, eight runs)
	Film-formation by spraying	Rechtschaffner Res V 2-level fractional design for four variables with center point
	Nanoprecipitation and nanospray-drying	Design model based on integrated-variance optimal design for surface response

Table S3. Experimental design observations for PVA/PVP/PEGdiacid-based hydrogel formulations.

Run	Factors			Responses		
	Crosslinker % w/w	Temperature °C	Time min	EWC% w/w	GF % w/w	Permeation % w/w
1	10	80	80			
2	7.5	115	50			
3	7.5	115	50			
4	5	115	90			
5	5	130	40			
6	10	150	80			
7	2.25	80	80			
8	10	80	20			
9	2.25	150	80			
10	5	115	50			
11	10	150	20			
12	2.25	150	20			
13	2.25	80	20			
14	5	70	50			
15	10	160	50			
16	5	115	50			
17	7.5	115	10			
18	1.15	115	50			
19	10	130	40			

Table S4. Freeze-drying procedure for the lyophilised wafers.

Time (min)	Temperature (°C)	Vacuum (mTorr)	Set
30	-40	600	Ramp
60	-40	600	Hold
30	-30	600	Ramp
60	-30	600	Hold
30	-20	600	Ramp
60	-20	600	Hold
30	-10	600	Ramp
500	-10	600	Hold
30	0	600	Ramp
60	10	600	Ramp
60	25	600	Ramp
600	25	600	Hold

Cell viability evaluation

MT4 cells were cultured at 37°C in a humidified atmosphere (≥95% RH) with 5% CO_2. The cell suspension was homogenised by carefully pipetting up and down multiple times to ensure even distribution. A 19 μL aliquot of the cell suspension was pipetted into a well of a plate, followed by the addition of 1 μL of Solution 13. The mixture was homogenised by pipetting several times, after which 10 μL was transferred into the chambers of the NC-Slide A8™. The slide was loaded onto the tray of the NucleoCounter® NC-3000™ (ChemoMetec A/S, Allerod, Denmark) and analysed *via* the "Viability and Cell Count Assay" protocol. The NucleoCounter® NC-3000™ software NucleoView was used to determine the total cell concentration (cells/mL) and viability (percentage). The software automatically compensated for the 5% dilution caused by the addition of Solution 13.

Virus titration

MT4 cells were subcultured every 3–4 days and seeded at approximately 6×10^5 cells/mL prior to the experiment. This seeding density was achieved by pelleting the cells (5 min, 410 g) and resuspending them in a specific volume of supplemented RPMI medium. The virus stock was titrated and diluted tenfold on two separate plates. A flat-bottomed, 96-well microtiter plate was filled with 100 μL of medium per well, and 25 μL of virus stock was added to the six middle wells (2B–G). The virus in wells 2B–G was serially diluted fivefold by transferring 25 μL from the 2nd column to the 3rd column *via* a multichannel pipette and mixing thoroughly. This dilution process was repeated across nine subsequent columns. Next, 50 μL of medium and 50 μL of MT4 cell suspension were added to each well. The outer rows (columns 1 and 12, rows A and H) were filled with 150 μL of medium without any cells or virus. The same procedure was performed with the tenfold diluted virus. The microtiter plates were incubated at 37°C in a humidified atmosphere (≥95% RH) with 5% CO_2 for 5 days.

After 5 days of incubation, the cells were examined under an Eclipse Ts2R inverted research microscope (Nikon Instruments Inc., New York, US; cofunded by Fondation Dormeur) for HIV-induced cytopathogenic effects (CPEs). A well was scored as positive if any trace of CPE was observed. The MTT assay was then performed as described previously. Briefly, 20 μL of tetrazolium salt solution (MTT, 7.5 mg/mL in 1× PBS) was added to all the wells, and the plate was incubated for 1 hour at 37°C with 5% CO_2. Subsequently, 150 μL of medium was removed from the wells (without disturbing the cells), and 150 μL of 6% Triton X-100 (v/v) in acidified isopropanol (0.6% HCl v/v in solvent) was added. The plate was left in the dark overnight to facilitate the dissolution of the formazan crystals. The amount of formazan produced was quantified spectrophotometrically at 550/690 nm *via* a Multimode plate reader EnSightTM (PerkinElmer, Shelton, US).
